# Overcoming the Blood–Brain Barrier: Multifunctional Nanomaterial‐Based Strategies for Targeted Drug Delivery in Neurological Disorders

**DOI:** 10.1002/smsc.202400232

**Published:** 2024-10-06

**Authors:** Callan D. McLoughlin, Sarah Nevins, Joshua B. Stein, Mehrdad Khakbiz, Ki‐Bum Lee

**Affiliations:** ^1^ Department of Chemistry and Chemical Biology Rutgers, The State University of New Jersey 123 Bevier Road Piscataway NJ 08854 USA; ^2^ Department of Regulatory Science Graduate School Kyung Hee University Seoul 02447 Republic of Korea; ^3^ Institute of Regulatory Innovation through Science (IRIS) Kyung Hee University Seoul 02447 Republic of Korea

**Keywords:** blood–brain barriers, glioma, multifunctional nanoparticles, nanoparticle‐based drug delivery systems, neurodegenerative diseases, neurological disorders, targeted drug deliveries

## Abstract

The development of effective therapies for neurological disorders is a growing area of research due to the increasing prevalence of these conditions. Some neurological disorders that are prevalent and remain difficult to treat are glioma, neurodegenerative disease, ischemic stroke, and traumatic brain injury. Subsequently, the therapeutic efficacy of small molecules, proteins, and oligonucleotides remains a challenge due to the presence of the blood–brain barrier (BBB), a highly selective semipermeable membrane. To this end, multifunctional nanomaterials have emerged as promising vehicles for targeted drug delivery to the brain, due to their ability to transport therapeutics across the BBB selectively. The design of advanced nanomaterial‐based drug delivery systems capable of overcoming the BBB is influenced by many factors, such as fabrication technique and surface modification. This review explores the diverse range of nanomaterials, including polymer, lipid, gold, magnetic, and carbon‐based nanostructures, capable of effectively passing the BBB. These materials cross the BBB via a variety of established transport mechanisms for targeted delivery of therapeutics to the brain. Moreover, the structure and function of the BBB are highlighted and the potential for nanotechnology to aid the treatment of neurological disorders based on their ability to undergo transcytosis into the brain is highlighted.

## Graphical abstract

This review explores the diverse range of nanomaterials, including polymer, lipid, gold, magnetic, and carbon‐based nanostructures, capable of effectively passing the blood–brain barrier (BBB). Moreover, the structure and function of the BBB in both healthy and diseased states are discussed and the potential of nanotechnology in bypassing the BBB to ultimately treat neurological disorders is highlighted.

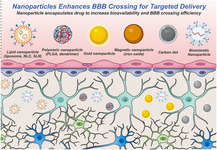

## 1 Introduction

Delivering therapeutic molecules across the blood–brain barrier (BBB) is one of the most significant obstacles in treating neurological disorders. The central nervous system (CNS) comprises the brain and spinal cord, while the peripheral nervous system regulates muscle movements and regulatory functions.^[^ ^1^ ^]^ The pathology of neurological disorders occurs in the brain which is protected by the BBB, limiting avenues for treatment. Factors like the BBB and P‐glycoprotein pump transporter restrict drug delivery across the brain. Typically, lipid‐soluble drugs smaller than 400 Da or have less than 8 hydrogen bonds can freely pass the BBB through transmembrane diffusion, making them ideal for delivery.^[^ ^2^, ^3^ ^]^ In general, BBB permeation decreases 100‐fold as the MW of a drug is increased from 300 to 450 Da. The mechanism of this MW threshold phenomenon is consistent with the formation of transient water pores in biological membranes, typically caused by transient fluctuations in fatty acyl side chain conformation in membrane lipids. Furthermore, Stein et al. observed a tenfold decrease in membrane permeation with each pair of hydrogen bonds on the solute, which has since been confirmed in studies of in vivo BBB transport of steroid hormones and oligopeptides.^[^ ^4^ ^]^ Past the BBB, therapeutics often need to specifically target cell types like neurons to treat the disease effectively.

Moreover, the behavior and characteristics of the BBB are strongly influenced by the delicate microenvironment of the brain. As a result, neurological disorders frequently cause significant changes in the structure and function of the BBB, which are crucial to disease pathology. In the presence of brain tumors, the functionality of the BBB is significantly altered, partially due to angiogenesis, causing the BBB to be known as the blood–brain–tumor barrier (BBTB). The BBTB only exists in the presence of brain tumors, so it is not of interest when targeting neurodegenerative disease, stroke, and traumatic brain injury (TBI).^[^ ^5^, ^6^ ^]^ Drug delivery to brain tumors is limited by three different barriers: the BBB, the blood–cerebrospinal–fluid barrier, and the plasma membrane of the tumor cells. Treatment of glioma and malignant brain metastases is limited by both the BBB and the BBTB. The BBTB forms close to the tumor site and is thought to be a main cause of low systemic drug delivery efficacy to the site of brain metastases.^[^ ^5^, ^7^, ^8^ ^]^ A primary barrier to treating most neurodegenerative diseases is BBB passage due to the low delivery capacity of many potential therapeutics past the BBB to the target of interest.^[3a]^ Numerous clinical trials for neurodegenerative disease therapeutics have been unsuccessful due to their minimal barrier passage, leading to high dosage requirements.^[^ ^9^ ^]^ In addition to difficulty passing the BBB with therapies, a key contributing factor to toxic compound accumulation and dysfunction of normal pathways is regulated by BBB disruption. The influx of toxic compounds due to BBB disruption occurs in many diseases, including but not limited to Alzheimer's disease (AD), Parkinson's disease (PD), ischemic stroke, and TBI.^[^ ^10^ ^]^

**Figure 1:**
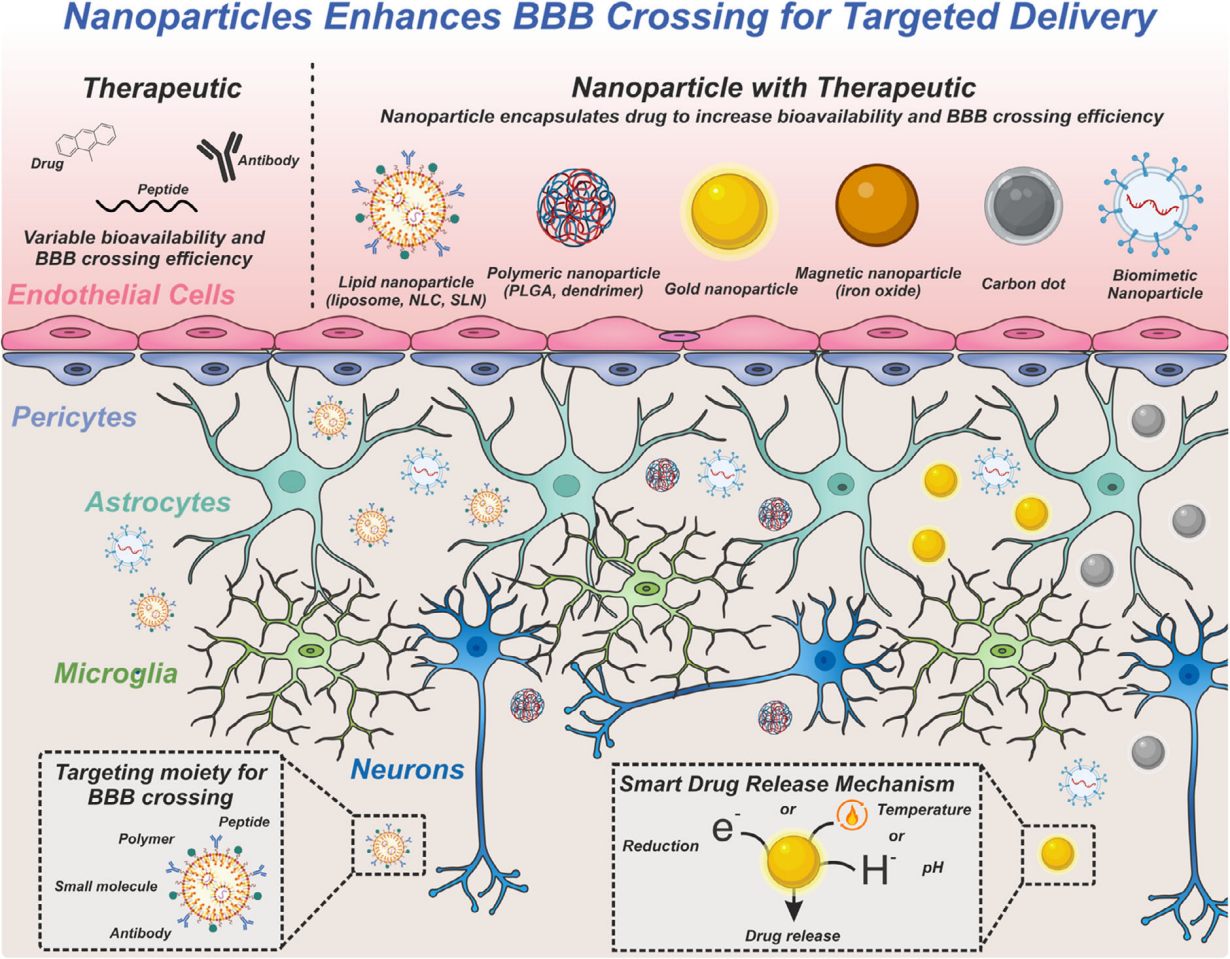
Nanoparticles enhance BBB crossing for targeted delivery. Nanoparticles can be formulated to leverage the mechanisms of BBB passage to target diseases in the brain.

Multifunctional nanosystems have substantial promise in treating neurological disorders due to their ability to selectively target tissues and cells of interest as well as their multifunctional capabilities based on the material and size.^[3a]^ The thoughtful design of nanoparticles (NPs) is critical for the passage of the BBB and specific targeting of the disease site. Organic and inorganic nanomaterials, including polymer‐based, lipid‐based, gold, and carbon‐based nanomaterials, have demonstrated significant potential in targeting diseases by noninvasively bypassing the BBB (**Figure** **1**).^[^ ^11^ ^]^ Magnetic NPs (MNPs) have also been shown to pass cell barriers.^[^ ^12^ ^]^ Physical parameters, like magnetic properties and particle size, field strength and geometry, and physiological parameters, like the depth to target, the blood flow rate, vascular supply, and body weight, are involved in the design of magnetic drug delivery systems.^[^ ^12^ ^]^ As mentioned, the treatment of diseases related to the CNS and brain faces many challenges due to cellular barriers that limit drug delivery to the brain and prevent the desired drug concentration from accumulating in the target tissue.

The main categories of transport across the BBB are passive and active transport. Methods for active transport are highly sought after because they target nanomaterials to the BBB, while passive transport lacks targeting and passes the BBB by coincidence.^[^ ^13^ ^]^ Additionally, size and charge of NPs are crucial factors for crossing the BBB. NPs with a diameter of up to 70 nm have been shown to cross the BBB with limited off‐target effects. In the context of surface charge, cationic NPs pass the BBB more efficiently than neutral or anionic NPs, but neutral and anionic NPs tend to exhibit less neurotoxicity than cationic ones.^[^ ^14^ ^]^ To this end, the accumulation of NPs in the brain can lead to toxicity.^[^ ^15^, ^16^ ^]^ When designing nanotherapeutics, only NPs with carefully controlled quantities and specific physicochemical properties should be permitted to cross the BBB to minimize this risk.^[^ ^15^, ^16^ ^]^ Researchers must design NPs with the appropriate characteristics to ensure safer and more effective nanomaterial‐based drug delivery systems for treating neurological disorders.^[^ ^15^, ^16^ ^]^ In this article, drug delivery systems that can overcome this barrier to treat glioblastoma, neurodegenerative diseases, ischemic stroke, and TBI have been reviewed. Also, for a better understanding of the penetrating mechanism and its evaluation in vivo, a brief review of BBB structure in healthy and diseased microenvironments is discussed.

## 2 Structure and Function of the BBB in Healthy and Diseased Brains

**Figure 2:**
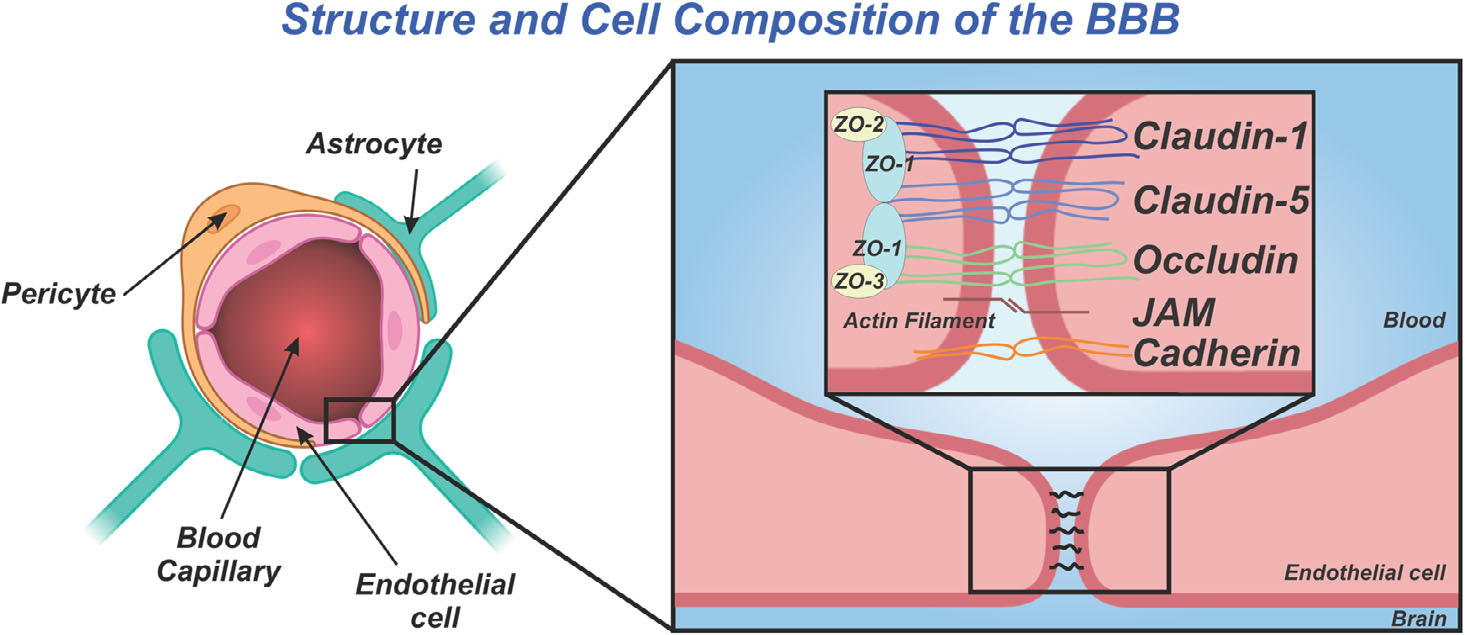
BBB structure and junctions that prevent passage in a healthy brain. Cell types and important proteins relevant to tight junctions and adherens junctions are labeled.

The BBB protects the microenvironment of the brain for functional synaptic signaling pathways that control motor skills, memory, and many more functions from outside influences.^[^ ^17^ ^]^ Designing an ideal material for brain therapeutics requires BBB passage, while mitigating off‐target effects. Other important considerations include even distribution inside the brain, cell entry, target selection, and exocytosis from cells and the brain after therapeutic function. The BBB is critical to maintaining proper homeostasis in the brain. It is composed of endothelial cells that surround cerebral capillaries and microvessels and are in close contact with astrocytes and pericytes. The space that endothelial cells meet is composed of tight junctions (TJs) and adherens junctions (AJs), which create a barrier surrounding the brain (**Figure** **2**).^[^ ^18^ ^]^ Astrocytes surround 99% of the abluminal surface of the brain capillary and neuronal endings.^[^ ^19^ ^]^ In neurodegenerative disease, stroke, and TBI, the BBB is often disrupted, giving entry to toxic compounds and leading to changes in normal metabolic pathways that cause many impairments in the function of astrocytes, neuron cells, and microglia cells.^[^ ^20^ ^]^ Understanding the BBB structure is critical to designing NP‐based systems for treating diseases.

### 2.1 Endothelial Cells and Tight Junctions

Endothelial cells are the core structure for the BBB and form a physical barrier between blood and the brain. TJs and AJs form at the gap between endothelial cells and allow the passage of select species to regulate the BBB microenvironment.^[^ ^21^ ^]^ The primary obstacle to passage through the BBB is the paracellular transport of hydrophilic molecules across the barrier.^[^ ^22^ ^]^ TJ integral proteins, including occludin, claudin, and tetraspanin, are made of four transmembrane regions, each consisting of two extracellular domains with amino and carboxyl‐terminal ends, and are predicted to be oriented towards the cytoplasm. Each protein has a unique structure, made of two extracellular loops and an intracellular region with amino‐ and carboxyl‐terminated groups, which is important for regulating molecules through TJs.^[^ ^23^ ^]^ Occludin extracellular loops are approximately the same size, while claudin and tetraspanin extracellular loops vary in size. They are composed mainly of tyrosine and glycine, while claudin has several charged residues that are thought to affect ion transportation via paracellular space.^[^ ^23^ ^]^ TJs are also composed of junctional adhesion molecules (JAMs). Several cytoplasmic accessory proteins, including zonula occludin protein 1 (ZO‐1), ZO‐2, and cingulin, modulate paracellular transport in response to different stimuli.^[^ ^24^ ^]^ The interactions between the transmembrane proteins vascular endothelial cadherin (VE‐cadherin) and epithelial cadherin (E‐cadherin) cause AJ formation.^[^ ^25^ ^]^ While AJs are less studied than TJs, AJs are important for initiating cell–cell interactions that lead to TJ formation and play a role in retaining the structure of the BBB. VE‐cadherin has also been shown to be integral in organizing vasculature and endothelial cell assembly into the BBB.^[^ ^26^ ^]^

### 2.2 Pericytes, the Basement Membrane, and Astrocytes

Pericytes, another main cellular component of the BBB, play a crucial role in maintaining BBB integrity, angiogenesis, regulation of cerebral blood flow, clearance of cellular debris, and generation of pluripotent cells.^[^ ^27^ ^]^ Pericytes play a vital role in pathology and maintaining the microenvironment inside of the BBB. In ischemic stroke, pericytes trap blood cells and prevent microcirculatory reperfusion after clot removal. However, some recent papers deny the function of pericytes in regulating brain blood flow.^[^ ^28^ ^]^ Endothelial cells and pericytes are key to basement membrane production, which encompasses the noncellular material in the brain. It is extremely complex and, therefore, less studied than the other components of the BBB.^[^ ^29^ ^]^ The function of the basement membrane changes during ischemic stroke.^[^ ^30^ ^]^ There is a dynamic interaction between pericytes and their surrounding basement membrane due to the role of pericytes in the direct and indirect deposition of basement membrane matrix proteins.^[^ ^31^ ^]^

Astrocytes are a type of glial cell that have been identified to play a crucial role in maintaining the BBB. They are the reason for the adaptive plasticity of the nervous system and are essential for functional regeneration after injury.^[^ ^32^ ^]^ Astrocytes are branched cells with a diameter and volume of 40–60 μm and 6.6 × 10^4^ μm^3^, respectively. Branches, branchlets, and leaflets occupy about 90–95% of the area in astrocytes. Astrocyte morphology, number of synapses, and proximity of astrocyte leaflets to synapses differ in each brain region. These features are also affected by injury and illness.^[^ ^33^ ^]^ There is evidence that astrocyte foot ends play a role in BBB formation and are essential to TJ formation.^[^ ^34^ ^]^ Astrocytes also release a number of neurotrophic factors that are indicated to be critical in signaling to maintain BBB function. Changes in astrocyte function are reported to affect BBB permeability and TJ formation. Additionally, the interactions between pericytes and astrocytes are crucial for vasculogenesis and overall BBB maintenance.

### 2.3 BBB Malfunction in Diseases

During the early stages of most neurological disorders, the BBB typically starts to malfunction. This opens the gate for many neurotoxic species to enter the brain, which in turn causes substantial changes in multiple pathways that are normally responsible for maintaining a healthy BBB microenvironment. Additionally, varying degrees of BBB disruption are observed among patients with the same disease. Different diseases are correlated to varying changes in levels of important molecules, ions, and cells, as well as the dysregulation of important pathways. For example, claudin proteins in TJs play a role in dysregulating the BBB in AD, PD, and ischemic stroke. Changes to AJs also lead to a large disruption of the BBB.^[^ ^35^ ^]^ Due to the complex interactions involved in BBB disruption, determining the timeline and mechanism of disruption has been challenging. Barrier disruption stems from changes in the pathology of important mechanistic pathways and causes the accumulation of toxic compounds that create reactive oxygen species (ROS) and inflammation. These changes in BBB function require further study to create therapies capable of targeting neurodegenerative diseases.^[^ ^36^ ^]^ Neurodegenerative diseases are highly connected to the cerebellum because of their importance in cognitive processes, and the diseases link to visual and auditory cortexes.^[^ ^37^ ^]^ Apolipoprotein E (ApoE) is an essential category of lipoproteins that aid in cholesterol transport by transporting lipids across brain cells using ATP‐binding cassette transporters like ABCA1. ApoE levels are also highly correlated to BBB dysfunction in many diseases, including AD and TBI.^[^ ^17^, ^38^ ^]^ GLUT1, part of the glucose transporter protein family, selectively shows up in endothelial cells in the BBB and is linked to the passage of neuroactive drugs through the BBB. GLUT1 expression is downregulated in many neurodegenerative diseases, ischemic stroke, and TBI, leading to BBB dysfunction as well.^[^ ^39^ ^]^

#### 2.3.1 Glioblastoma Multiforme

Glioblastomas are the most common and deadly form of brain cancer, as they are grade IV gliomas.^[^ ^40^ ^]^ Gliomas encompass 80% of all primary brain tumors, and glioblastomas encompass 54% of all glioma tumors.^[^ ^41^ ^]^ BBB damage and the resulting BBTB that protects cancer cells is a central hallmark of malignant brain cancers.^[^ ^6^ ^]^ The BBB loses its integrity and has an increase in permeability with the formation of the BBTB. However, it is still extremely difficult for therapeutics to cross the BBTB due to certain aspects that are retained, such as efflux transporters that are a limiting factor in healthy BBB passage of many therapeutics as well. Understanding the mechanisms underpinning BBB remodeling into the BBTB and developing targeted therapeutics is pertinent for improving the survival rate of glioma patients and lowering the off‐target effects of drugs currently used.^[^ ^6^, ^42^ ^]^ Although there is selective BBB disruption in glioblastoma, altered BBB expression is linked to the influx of metastatic cells into the brain and is thought to be a large part of the reason many drugs struggle to pass the BBB for targeting glioblastomas.^[^ ^43^ ^]^ Vascular malformations disrupt normal blood flow to the brain, resulting in leaky and dysfunctional blood vessels with increased fluid pressure that can be correlated with increasing brain tumor volume and is an important part of BBB malfunction.^[^ ^44^, ^45^ ^]^ Another study, looking at the effect of BBB disruption on innate and adaptive immune responses due to a glioblastoma, showed that the number of dendritic cells significantly increased in the murine glioblastoma model. Furthermore, there was an upregulation of CD86, which plays a role in immune recognition of glioblastoma cells.^[^ ^46^, ^47^ ^]^ BBB composition and pressure changes lead to increased challenges in the delivery of therapeutics to brain tumors. BBB disruption is sporadic in brain tumors, and the tumor is often protected by intact and remodeled portions of the BBB.^[^ ^48^ ^]^ For this reason, mimicking BBTB in vitro is extremely important.

#### 2.3.2 Neurodegenerative Disease

AD is the most prevalent neurodegenerative disease and affects an increasing percentage of the population.^[^ ^49^ ^]^ The annual mortality of neurodegenerative diseases is estimated to be growing at a rate of 166% a year, and the 5 year survival rate for all patients with malignant brain tumors was 36% between 2009 and 2015.^[^ ^42^, ^50^ ^]^ It has been reported that while amyloid beta (Aβ) expression was elevated in animals lacking ABCA1, ApoE levels were downregulated.^[^ ^17^ ^]^ ApoE4, a specific lipoprotein, has also been linked to the downregulation of expression and disruption of the BBB. The mechanisms behind this are not known yet, but there is hope that by creating models of the BBB, there will be further insight into the correlation between ApoE4 levels and endothelial BBB disruption.^[^ ^17^ ^]^ ApoE levels have been shown to be highly increased in patients with mild cognitive impairment and other forms of dementia, and those levels start to decline rapidly when going from mild cognitive impairment to Alzheimer's dementia patients, as the neurons have already died at these late stages in the disease, so ApoE levels are also extremely downregulated. ApoE levels are also correlated with the loss of pericytes in AD, which is heavily connected to acceleration in the development of Aβ and tau, two of the main targets in the disease pathology.^[^ ^36^ ^]^ A decline in claudin levels has a major impact on the proper formation of the BBB.^[^ ^51^ ^]^ ROS plays an extremely important role in neuronal cell death through downstream processes such as an increase in IL‐1B expression and ASK1 activation.^[^ ^52^ ^]^ GLUT1 downregulation in Alzheimer's leads to cerebral microvascular degeneration and BBB breakdown.^[^ ^53^ ^]^

PD is a neurodegenerative disease that presents as a proteinopathy, primarily through the aggregation and fibrillation of alpha‐synuclein protein, and is frequently characterized by dopaminergic neurodegeneration. The process of fibrillation is toxic and leads to selective neuronal death in the substantia nigra pars compacta. The disruption of the BBB is relevant to the pathogenesis of PD since toxic species disturb the homeostasis of the brain microenvironment when leakage occurs.^[^ ^54^ ^]^ ApoE levels in PD have been shown to be extremely upregulated. Increased lipoprotein vesicle levels and uptake of vesicles into dopaminergic neurons are also increased, which is linked to alpha‐synuclein spreading through cells and changes in the brain microenvironment. In 2021, utilizing a PD mouse and an in vitro BBB model made of three different cell types, Lan et al. showed that there was a decreased expression of TJ proteins, increased vascular permeability, and accumulation of oligomeric alpha‐synuclein in astrocytes in the brain.^[^ ^55^ ^]^ The role of astrocytes in the BBB has continued to be redefined in recent years, and this study shows the key role astrocytes play in BBB malfunction. Alpha‐synuclein accumulation in activated astrocytes was due to the expression and release of vascular endothelial growth factor A (VEGFA) and nitric oxide from oligomeric alpha‐synuclein. Blocking the VEGFA signaling pathway protected the barrier against the harmful effects caused by oligomeric alpha‐synuclein in the PD mouse model. Increased expression of nitric oxide and VEGFA was also found in the brains of PD patients through this study.

Huntington's disease (HD) is characterized by pathogenic cytosine‐adenine‐guanine (CAG) nucleotide repeats of at least 40 in size, which are key to disease pathology as well as abnormal expression of the Huntington protein (HTT). Notably, HTT has a propensity to accumulate and aggregate to form intranuclear and intracytoplasmic neuronal inclusions. There is evidence of alterations in the BBB structure and function in HD mouse models and patients, such as an increase in blood vessel density, a reduction in blood vessel diameter, and an increase in BBB leakage.^[^ ^56^ ^]^ Very early in the disease, before the onset of symptoms, defections in TJs start in HD, as evidenced in mouse models.^[20a]^ BBB malfunction in HD also stems from homeostasis of the brain‐derived neurotrophic factor, defective histone acetylation, and sphingosine‐1‐phosphate (S1P), a type of sphingolipid that is connected to neurite growth and microglial survival.^[^ ^57^ ^]^ Huntington pathology has been linked to Wnt signaling, which is a factor in BBB leakage.^[^ ^58^ ^]^

#### 2.3.3 Ischemic Stroke

Ischemic strokes encompass 86% of all strokes. They are characterized by loss of oxygen and glucose delivery to the brain and increased endothelial ion transporter activity, leading to higher secretion of sodium and chloride ions across the BBB and higher calcium levels inside the BBB.^[^ ^59^ ^]^ Strokes are the leading cause of death in the elderly worldwide.^[^ ^60^ ^]^ The increase in calcium levels leads to ROS production through mitochondria dysfunction. Inflammation in ischemic stroke is also highly connected to changes in other inflammatory mediators, such as cytokines.^[^ ^61^ ^]^ The claudin proteins involved in TJs are remodeled in ischemic stroke to allow macromolecules to permeate the BBB, which is the main reason for disease pathology.^[^ ^35^ ^]^ Homozygous ApoE4 levels have been highly expressed in pre and postischemic stroke dementia patients, while heterozygous ApoE3/ApoE4 has low expression levels. It has been linked to blood‐brain dysfunction, as ApoE4 damages blood vessels.^[^ ^62^ ^]^ The role of abnormal expression of long noncoding RNAs has been increasingly studied in recent years due to their role in ischemic stroke.

#### 2.3.4 Traumatic Brain Injury

Generally, the severity of TBI has been classified as mild, moderate, and severe by a universal classification system (Glasgow Coma Scale, GCS).^[^ ^63^ ^]^ Severe TBI, in particular, exhibits a significant probability of hypoxemia, hypotension, and brain swelling.^[^ ^64^ ^]^ Hypoxemia remains explicitly one of the most common symptoms of severe TBI and can significantly exacerbate the onset of secondary brain injury.^[^ ^65^ ^]^ In the context of TBI, pathological alterations to the BBB structure result in leakiness inside and around the cortical lesion in addition to the hippocampus and hypothalamus. This BBB dysfunction leads to proteins and fluids leaking out and immune cell migration out of the brain rather than allowing certain toxic species to enter the brain.^[^ ^66^ ^]^ Some levels affected by BBB disruption include changes in calcium levels and increased release of chemicals such as excitotoxin, inflammatory mediators, and oxidative factors. The damaged state of the BBB has been investigated for potential pathways of entry to the brain for TBI therapy. To mimic the BBB pathways in disease microenvironments, an understanding of mutations to natural mechanisms in disease is required. ApoE is also crucial in TBI due to its influence on the nuclear factor kappa B (NF‐κB) and the matrix metalloproteinase‐9 (MMP‐9) pathway. ApoE levels are highly correlated to the effects of TBI, including a correlation with BBB disruption.^[^ ^38^ ^]^ MMP‐9 is a group of proteolytic enzymes that bind to zinc, and their role is to remodel the extracellular matrix. At the same time, diseased microenvironments can help degrade parts of the basal lamina, such as fibronectin, collagen, and laminin. Downregulation or suppression of NF‐κB leads to reduced MMP‐9 levels, which can help alleviate BBB disruption in TBI.

## 3 NP‐Mediated Transcytosis Across the BBB for the Treatment of Diseases

**Figure 3:**
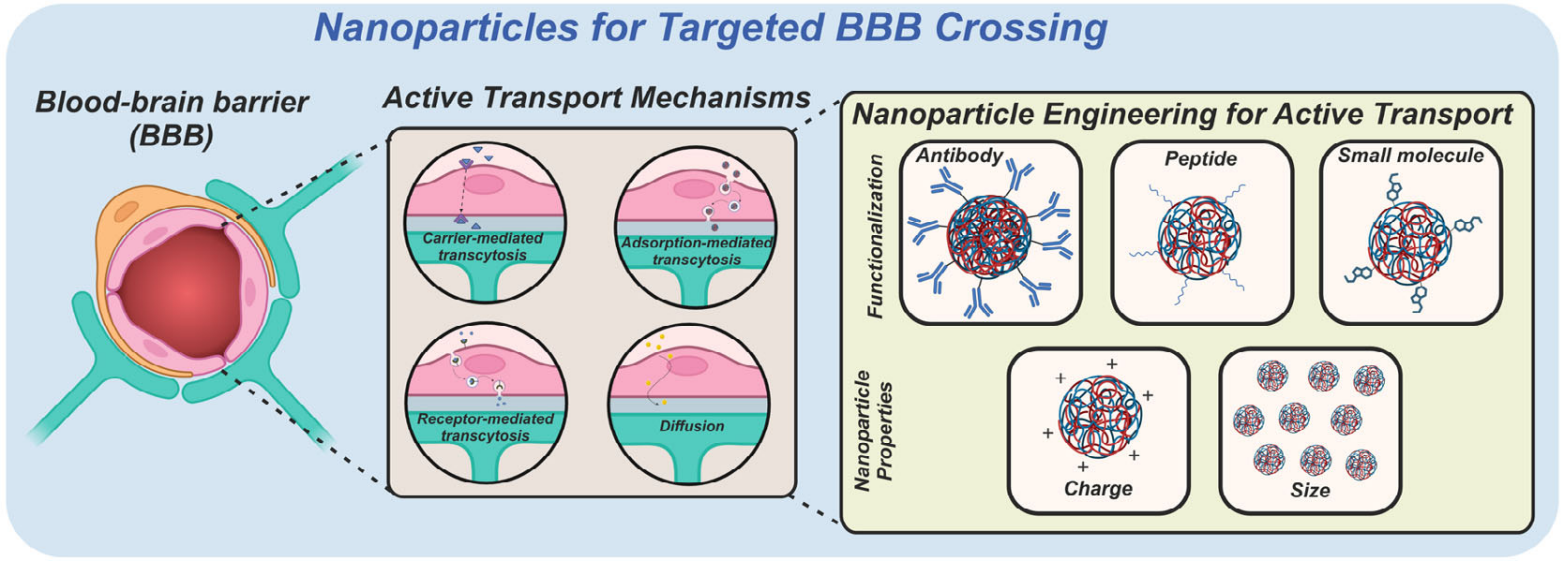
Various mechanisms of transcytosis across the BBB in a healthy brain. Each overlay bubble portrays one mechanism of transport from endothelial cells to the basement membrane.

Recent advances in materials science and biomedical engineering have led to significant progress in the formulation of nanomaterial‐based strategies for treating cancer, neurodegenerative disease, ischemic stroke, and TBI. The intrinsic properties and tunability of nanomedicine improve the targeting of tumor cells by offering increased drug solubility, extended retention time and stability in the body, selective targeting, and reduced side effects associated with more potent treatments.^[^ ^67^ ^]^ Most interestingly, nanomedicine has offered an unprecedented ability to facilitate drug delivery across the BBB, granting access to disease‐associated brain regions.^[^ ^68^ ^]^ For most small‐molecule drugs, diffusion across the BBB is the most straightforward transport mechanism, utilizing a concentration gradient through which certain lipid‐soluble molecules can pass. Additionally, this method of BBB transcytosis relies on passive targeting of the BBB, decreasing the overall localization in the brain. In the context of NPs, certain characteristics are typically emphasized for active transcytosis through the BBB, utilizing one or more of three mechanisms: 1) adsorption‐mediated transcytosis (AMT), 2) carrier‐mediated transport (CMT), and 3) receptor‐mediated transcytosis (RMT). NP‐mediated BBB transcytosis is preferred over paracellular transport, as the tightly fused junctions of the cerebral endothelium essentially form a continuous lipid layer that allows passage of only small, electrically neutral, lipid‐soluble molecules. In this way, paracellular transport is practically inaccessible to NPs. RMT, AMT, and CMT have greater BBB targeting ability, as they are all active modes of transport (**Figure** **3**). CMT utilizes a group of membrane transport proteins to transport polar molecules, such as sugars and amino acids, into endothelial cells through their corresponding transmembrane proteins.^[^ ^69^ ^]^ An example of CMT across the BBB is glucose, mediated by glucose transporter type 1 (GLUT1). Larger molecules are mainly transported via RMT, a popular mechanism for NP transportation across the BBB.^[14a]^ RMT is induced via peptide receptors on cell membranes, mediating the transport of the associated ligand.^[^ ^69^ ^]^ Furthermore, RMT is a bidirectional mechanism—both blood‐to‐brain and brain‐to‐blood transport can occur. Mediated by clathrin‐dependent endocytosis, AMT uses electrostatic interactions between cationic molecules and the luminal surface of endothelial cells to perform transcytosis.^[^ ^70^ ^]^ Unlike RMT, AMT is unidirectional, transporting from blood to the brain.

Cationic NPs are a classic example of NPs that utilize AMT, as they facilitate passage without specific targeting of a BBB receptor. However, cationic NPs have a propensity for disrupting cellular membranes and, subsequently, the BBB, potentially compromising its structural integrity.^[^ ^71^ ^]^ Additionally, particle size tends to be a limiting factor for BBB transcytosis, as smaller particles can interact with the BBB without major disruption. For each of the three primary mechanisms of BBB transcytosis, the correlation between NP size and BBB structural disruption is evident.^[^ ^72^ ^]^ Deviating from AMT, conjugation of BBB receptor‐ligands has remained a common and effective method to increase BBB transcytosis for NPs due to the specificity of the interaction, prioritizing RMT as the primary mechanism. Finally, in the context of BBB‐penetrating nanotherapeutics, the accurate design and synthesis of biodegradable NPs are requested, as it is critical for restricting the build‐up and cytotoxicity of delivered NPs to the targeted brain region. Various nanobased delivery systems have been developed, including polymer, lipid, gold, magnetic, and carbon‐based NPs. Each type and composition of nanomaterial has unique properties that facilitate the treatment of glioblastoma, neurodegenerative diseases, ischemic stroke, and TBI. A list summarizing the materials previously used in literature to target and pass the BBB is shown in **Table** **1**.

**Table 1:** Classes of NPs for BBB transcytosis with associated mechanism of transport, targeting mechanism, and mechanism for drug‐release for the associated brain‐related disease.

Class of materials	Type of pathological BBB	In vitro and in vivo testing methods	Vehicle	Vehicle properties for BBB crossing	BBB targeting mechanism	Therapeutic molecule	Drug release mechanism	References
Polymer	Alzheimer's disease	AD‐like transgenic mice	Biodegradable PEGylated PNP	Anti‐amyloid‐beta	RMT via LRP‐1 or P‐gp	Doxorubicin	Conjugated antibody activty	Carradori et al.^[^ ^79^ ^]^
Polymer	Glioblastoma	BALB/c Nude mice	Poly(vinyl alcohol) nanogel	Cyclic RGD peptide	RMT	Taxol/paclitaxel	Reduction‐responsive	Chen et al.^[^ ^76^ ^]^
Polymer	Glioblastoma	hCMEC/D3 and T98G, 3D Spheroid, mice	PLGA	L‐carnitine	Carrier‐mediated transport; Na+‐coupled carnitine transport 2 (OCTN2)	Paclitaxel	Passive	Kou et al.^[^ ^75^ ^]^
Polymer	Glioblastoma	bEnd.3 cell	dCatAlb‐pDNP	Cationic albumin	AMT	Doxorubicin	pH‐responsive	Muniswamy et al.^[^ ^73^ ^]^
Polymer	TBI	RAW 264.7; pediatric TBI rabbit	Dendrimer	Surface conjugation of the drug	AMT	Sinomenine	Conjugated drug activity	Sharma et al.^[^ ^93^ ^]^
Polymer/lipid	Glioblastoma	SCID mice	Polysorbate 80/poly(methacrylic acid)/starch	Polysorbate80 (LDL mimic)	RMT	DTX	pH‐responsive	He et al.^[^ ^182^ ^]^
Polymer	Glioma	GL261‐Luc2 tumor bearing C57BI/6 albino mice	PLGA NP	Rabies virus glycoprotein (RVG29)	RMT	Chemotherapeutic captothecin		Cook et al.^[^ ^183^ ^]^
Polymer	Neurodegenerative disease	Adult male Swiss CD‐1 mice	PLGA NP	Antibody targeting transferrin	RMT	Loperamide	Passive	Fornaguera et al.^[^ ^83^ ^]^
Polymer	Glioblastoma	Glioma‐bearing mice	PEG/PCL NPs	Angiopep‐2; activatable cell penetrating peptide	RMT	DTX	MMP responsive cell penetrating peptide	Gao et al.^[^ ^184^ ^]^
Polymer	Neurodegenerative disease	hCMEC/D3 cell line	Poly ethylcyanoacrylate NPs	Polysorbate80 (LDL mimic)	RMT	Andrographolide	Passive	Guccione et al.^[^ ^185^ ^]^
Class of materials	Type of pathological BBB	In vitro and in vivo testing methods	Vehicle	Vehicle properties for BBB crossing	BBB targeting mechanism	Therapeutic molecule	Drug release mechanism	Reference
Polymer	Ischemic Stroke	Ischemic mice	PEG/PCL/enzyme cleavable peptides	AMD3100 ‐ CXCR4 antagonist	RMT	Glyburide	Protease‐responsive/enzyme‐cleavable	Guo et al.^[^ ^89^ ^]^
Polymer	Glioblastoma	Mice bearing intracranial C6 glioma	PEG/PLA NPs	F3 peptide	Positive charge; diffusion	Paclitaxel	Passive	Hu et al.^[^ ^186^ ^]^
Polymer	AD	Porcine brain capillary endothelial cells (PBCECs)	PLGA	Anti transferrin receptor monoclonal antibody (OX26)	RMT	Peptide iAβ5	Passive	Loureiro et al.^[^ ^187^ ^]^
Polymer	AD	Kunming mice	PLGA	Lactoferrin conjugated trimethylated chitosan	RMT	Huperzine A	Passive	Meng et al.^[^ ^188^ ^]^
Polymer	AD	SK‐N‐SH	PEG/PLA NPs (varying amounts of PEG)		Passive	Curcumin	Passive	Rabanel^[^ ^189^ ^]^
Polymer	Ischemic Stroke	Adult male Wistar rats	Polymeric micelle (PEO‐PPO‐PEO)	Pluronic block copolymers	Passive	Nimodipine	Passive	Sotoudegan et al.^[^ ^190^ ^]^
Polymer	Glioblastoma	BCEC/C6 glioma cells	PLGA core Lipid shell (DSPE‐PEG/Lecithin)	Angiopep‐2 (Brain targeting)/aptamer AS1411 (Glioma targeting)	RMT	Doxorubicin	Passive	Wang et al.^[^ ^191^ ^]^
Polymer	Neurodegenerative disease	Sprague‐Dawley rats	PAMAM dendrimer	Cationic dendrimer	AMT	Donepezil	Conjugated drug activity	Singh et al.^[^ ^192^ ^]^
Polymer	Glioma	Wistar rats	PAMAM‐chitosan conjugate	Intraperitoneal delivery	AMT	Temozolomide	Passive/degradation‐based release	Sharma et al.^[^ ^193^ ^]^
Polymer	Glioblastoma	BALB/c mice and CB‐17 SCID mice	Poly(amidoamine) dendrimer‐based carrier	Angiopep‐2/EGFR targeting peptide	RMT/glioblastoma targeting	Doxorubicin	pH‐responsive	Liu et al.^[^ ^194^ ^]^
Polymer	Glioblastoma	BMVECs transwell; C6 glioma cells	PEG‐PAMAM‐WGA‐Tf	Transferrin/wheat grass agglutinin	RMT	Doxorubicin	pH sensitive linker for release	He et al.^[^ ^195^ ^]^
Polymer	Glioblastoma	HBMEC/C6 glioma transwell	PAMAM dendrimer	Angiopep‐2	RMT	Borneol	pH‐dependent	Han et al.^[^ ^196^ ^]^
Polymer	Glioma	Albino rat	PAMAM‐BBR	Cationic dendrimer	AMT	Berberine	Conjugated drug activity	Gupta et al.^[^ ^197^ ^]^
Polymer	Glioma	bEnd.3 cell and U373 MG cells	Polyether*‐co*‐polyester dendrimer	D‐glucosamine	RMT	Methotrexate	Diffusion	Dhanikula et al.^[^ ^198^ ^]^
Class of materials	Type of pathological BBB	In vitro and in vivo testing methods	Vehicle	Vehicle properties for BBB crossing	BBB Targeting mechanism	Therapeutic molecule	Drug release mechanism	References
Polymer	Alzheimer's disease	bEnd.3 cell transwell model	Poly(epsilon‐lysine) dendrimer	Cationic dendrimer	AMT	Flurbiprofen	degradation based release	Al‐Azzawi et al.^[^ ^199^ ^]^
Polymer	AD	Male APPswe/PS1dE9 (APP/PS1) and C57BL/6 mice	PLGA‐PEG NP	Spherical shape and PEGylated	Passive	MH	Degradative release	Sanchez‐Lopez et al.^[^ ^80^ ^]^
Polymer	HD	WT and R6/2 mice	PLGA NP	Heptapeptide g7	RMT	Cholesterol	Degradative release	Birolini et al.^[^ ^87^ ^]^
MOF	Glioblastoma	BALB/c mice	Rabies virus biomimetic metal organic framework		RMT	Oxaliplatin	pH‐dependent	Qiao et al.^[^ ^200^ ^]^
MOF	Ischemic Stroke	HBMEC/PC12 coculture transwell	ZIF‐8 MOF capped ceria NPs		Passive	Cerium oxide ROS scavenging	pH‐dependent	He et al.^[^ ^201^ ^]^
Magnetic	Inflammation	bEnd.3 cells	Magnetic PLGA/LNPs	TAT	RMT	Hesperiden, naringin, and GSH	Passive	Wen et al.^[^ ^202^ ^]^
Magnetic	Glioblastoma	GBM6‐luc tumor bearing nude athymic mice	Magnetic chitosan/peg NPs	Tumor targeting chlorotoxin	Carrier‐mediated transcytosis	O(6)‐methylguanine‐DNA methyltransferase		Stephen et al.^[^ ^203^ ^]^
Magnetic	Glioma	U87MG Glioblastoma	Dextran‐spermine NPs	Transferrin	RMT	Capecitabine	pH‐responsive	Ghadiri et al.^[^ ^204^ ^]^
Magnetic	Glioma	Orthopic brain‐bearing rats	Magnetic polydiacetylene NCs with 10,12‐pentacosadiynoic acid monomers	Lactoferrin and magnetic localization	RMT and magnetic localization	Curcumin/lactoferrin	Passive	Fang et al.^[^ ^205^ ^]^
Magnetic	Glioma	Orthopic glioma model in vivo	Magnetic PLGA NPs	Transferrin receptor binding peptide T7 and magnetic localization	RMT and magnetic localization	Paclitaxel and curcumin	Degradative release	Cui et al.^[^ ^206^ ^]^
Magnetic	Glioma	BALB/c mice	Magnetic silica PLGA NPs	Transferrin	RMT	Doxorubicin and paclitaxel	Passive	Cui et al.^[^ ^207^ ^]^
Magnetic	Glioma	9L‐glioma rat model	Starch and aminosilane coated MNPs	Magnetic localization		Hyperthermia	Intrinsic property	Cole et al.^[^ ^208^ ^]^
Magnetic	Alzheimer's disease	Tg2576 transgenic mice	Superparamagnetic iron oxide with PEG	Magnetic localization		Curcumin	Conjugated drug activity	Cheng et al.^[^ ^209^ ^]^
Class of materials	Type of pathological BBB	In vitro and in vivo testing methods	Vehicle	Vehicle properties for BBB crossing	BBB Targeting mechanism	Therapeutic molecule	Drug release mechanism	Reference
Magnetic	Glioma	Glioma bearing male Wistar rats	Folate conjugated magnetic triblock copolymer NPs	Folic acid	RMT	Temozolomide	Degradative release	Afzalipour et al.^[^ ^210^ ^]^
Magnetic	Glioma	Sprague‐Dawley rats	Magnetic liposome	Magnetic localization		Paclitaxel	Passive	Zhao et al.^[^ ^211^ ^]^
Magnetic	Glioma	hCMEC/D3 cell line	Magnetoliposomes	ApoE‐peptide	RMT	Doxorubicin	Passive	Skouras et al.^[^ ^212^ ^]^
Magnetic	Glioma	bEnd.3 cell and C8‐d1A(astrocytes) transwell	Thermosensitive liposome loaded with SPIONs and doxorubicin	P1NS and TN‐C	RMT	Doxorubicin	Hyperthermic release upon magnetic alternating current	Shi et al.^[^ ^213^ ^]^
Magnetic	CNS lymphoma	bEnd.3 cells transwell	Liposome loaded with SPION‐PVA	Anti‐CD20 (RTX)	RMT	RTX	Conjugated drug activity	Saesoo et al.^[^ ^214^ ^]^
Magnetic	PD	PD rats	Fe_3_O_4_‐NMD‐lips		Magnetic localization	Nimodipine	Passive	Ji et al.^[^ ^215^ ^]^
Magnetic	Glioma	hCMEC/D3 and glioma cell line U251	SiO_2_@Fe_3_O_4_ NP	TAT	Magnetic localization/RMT	MRI contrast agent	N/A	Zhao et al.^[^ ^216^ ^]^
Magnetic	HD	Adult Wistar rats	BSA‐PAH core‐shell NPs	Cationic shell	AMT	Kynurenic acid	Passive/Degradative release	Varga et al.^[^ ^217^ ^]^
Magnetic	Glioblastoma	hBMEC and U‐87MG glioblastoma cells	CoFe_2_O_4_@BaTiO_3_	Magnetic field and GBM targeting	RMT	MIA690 (growth hormone‐releasing hormone antagonist)		Stewart et al.^[^ ^218^ ^]^
Magnetic	Glioma	C6 glioma bearing rats	SPIO NPs with PEG phospholipid coating		Passive	Doxorubicin and indocyanine green		Shen et al.^[^ ^219^ ^]^
Magnetic	Glioblastoma	GBM‐bearing BALB/c nude mice	IONP‐PAI/PLGA	cRGD‐labeled brain tumor cell membranes	RMT	MRI contrast agent	N/A	Duan et al.^[^ ^127^ ^]^
Magnetic	PD	6‐OHDA‐induced PD mice	MNP‐labeled hADSCs	Magnetic localization		hADSCs	N/A	Kim et al.^[^ ^129^ ^]^
Magnetic	AD	AD transgenic mice	DSPE‐PEG Congo Red/phenylboronic acid‐coated MNP	N/A	Passive	Rutin	ROS‐responsive	Hu et al.^[^ ^130^ ^]^
Magnetic	Ischemic Stroke	Ischemic mice	PAMN (PLT membrane‐coated MNP	Magnetic localization		L‐arginine	NO production via L‐arginine	Li et al.^[^ ^134^ ^]^
Lipid	Glioblastoma	C57BL/6 mice	SLN	Angiopep‐2	RMT	DTX	Passive/Degradative release	Kadari et al.^[^ ^100^ ^]^
Class of materials	Type of pathological BBB	In vitro and in vivo testing methods	Vehicle	Vehicle properties for BBB crossing	BBB Targeting mechanism	Therapeutic molecule	Drug release mechanism	References
Lipid	TBI	TBI mice	LNDs	Varying size of nanostructure (30 > 80 nm)	Passive	N/A	N/A	Khalin et al.^[^ ^108^ ^]^
Lipid	Glioblastoma	U87MG	SLN	MA	RMT	ETP	Degradative release	Kuo et al.^[^ ^101^ ^]^
Lipid	Glioblastoma	NU/J male and female mice	Transferrin‐coated liposome	Transferrin	RMT	Doxorubicin/erlotnib	Passive	Lakkadwala et al.^[^ ^220^ ^]^
Lipid	Glioma	BMVECs/C6 glioma and BALB/c nude mice	Transferrin‐cell penetrating peptide‐sterically stabilized liposome	Transferrin	RMT	Doxorubicin	Degradative release	Liu et al.^[^ ^221^ ^]^
Lipid	Glioblastoma	Adult Sprague‐Dawley rats	CSLN	OX26 Mab	AMT and RMT	Baicalin	Degradative release	Liu et al.^[^ ^102^ ^]^
Lipid	AD	AD rat model	LDL‐mimic NC	Lactoferrin	RMT	Curcumin	Degradative release	Meng et al.^[^ ^222^ ^]^
Lipid	Brain metastasis breast cancer	MDDA‐MB‐231Br‐Luc cells injected into female athymic nude mice to metastasize	Liposomal irinotecan	N/A	Enhanced‐permeation and retention	Irinotecan (Active metabolite: SN‐38)	Degradative release	Mohammad et al.^[^ ^223^ ^]^
Lipid	Glioblastoma	bEND.3 cells; ICR mice with GSC	Curcumin‐loaded liposomes	P‐aminophenyl‐a‐D‐mannopyranoside	CMT	Curcumin/quinacrine	Degradative release	Wang et al.^[^ ^224^ ^]^
Lipid	Ischemic Stroke	Male Sprague‐Dawley rats	LNP from SPC	Passive	Passive	OEA	Passive sustained release by crystalized OEA	Wu et al.^[^ ^107^ ^]^
Gold	Glioblastoma	C6 cells and glioma bearing mice	Folic acid‐BSA AuNC	Folic acid and BSA	RMT	AuNC as radiosensitizer	N/A	Kefayat et al.^[^ ^114^ ^]^
Gold	Glioblastoma	Male nude mice with implanted U87 cells	Dithio‐PEG‐AuNP	EGF peptide and size dependency	RMT	Doxorubicin	pH Responsive	Feng et al.^[^ ^115^ ^]^
Gold	TBI	Male C57BL/6J mice	Au_24_Cu_1_ and Au_24_Cd_1_ Clusterzymers	Diffusion		ROS scavenging via GPx, CAT, and SOD‐like enzymatic activity		Liu et al.^[^ ^123^ ^]^
Gold	General CNS	Wistar rats	PEG coated AuNP	MRI‐guided focused ultrasound		N/A		Etame et al.^[^ ^112^ ^]^
Class of materials	Type of pathological BBB	In vitro and in vivo testing methods	Vehicle	Vehicle properties for BBB crossing		BBB Targeting mechanism		Therapeutic molecule
Gold	AD	bEND.3 cells; mice	AuNR with CeO_2_	Amyloid beta‐targeted inhibitory peptide	RMT	CeO_2_ and KLVFF (Amyloid beta peptide)		Ge et al.^[^ ^118^ ^]^
Gold	SCI	Sprague‐Dawley rats	Zinc‐doped AuNC	Diffusion		DHLA	Conjugated drug activity	Lin et al.^[^ ^122^ ^]^
Gold	PD	Adult male C57BL/6 mice	AuNC	Diffusion		Histidine and DHLA	Conjugated drug activity	Mahapatra et al.^[^ ^117^ ^]^
Gold	Ischemic Stroke	Ischemic stroke Wistar rats	AuNP	Passive		Modafinal/MSCs	MSC delivery and conjugated drug activity	Nazarian et al.^[^ ^121^ ^]^
Gold	AD	PBCE transwell model	AuNP	Amyloid beta specific peptide	RMT	Amyloid beta specific peptide (CLPFFD)		Ruff et al.^[^ ^116^ ^]^
Gold	Ischemic stroke	3D model with astrocytes, pericytes, endothelial cells, microglia, oligodendrocytes, and neurons	Ultrasmall AuNP	Diffusion		N/A		Sokolava et al.^[^ ^119^ ^]^
Gold	SCI	C2Hx rats	AuNP	Wheat germ agglutinin horse radish peroxidase (HRP)	AMT	Wheat germ agglutinin HRP and 1,3‐dimethylzanthine	Conjugated drug activity	Zhang et al.^[^ ^225^ ^]^
Gold	TBI	Male Wistar rats	AuNP	Passive		AuNP	N/A	Hunt et al.^[^ ^124^ ^]^
Carbon	Glioblastoma	Zebrafish embryo	CuCDs	Diffusion		Curcumin	N/A	Sharma et al.^[^ ^144^ ^]^
Carbon	PD	SH‐SY5Y cells	Sodium citrate CDs	Diffusion		Disaggregation of fibrils	N/A	Guerrero et al.^[^ ^147^ ^]^
Carbon	AD	Zebrafish larvae	Carbon nitride CDs	Diffusion		MH	N/A	Zhang et al.^[^ ^148^ ^]^
Carbon	Ischemic stroke	MCAO rats	Crinis Carbonisatus CDs	Diffusion		Crinis Carbonisatus	N/A	Zhang et al.^[^ ^152^ ^]^
Carbon	TBI	TBI mice	L‐lysine CDs	Diffusion		L‐lysine	N/A	Li et al.^[^ ^226^ ^]^
Biomimetic	Glioblastoma	Zebrafish and C6‐Luc glioma‐bearing mice	Neutrophil‐derived exosomes	Receptor‐mediated transcytosis		Doxorubicin	Release upon fusion	Wang et al.^[^ ^156^ ^]^
Biomimetic	Glioblastoma	Glioma‐bearing mice	RBC membrane‐coated NP	c(RGDyK) ligand	RMT	DTX	Release upon fusion	Chai et al.^[^ ^158^ ^]^
Class of materials	Type of pathological BBB	In vitro and in vivo testing methods	Vehicle	Vehicle properties for BBB crossing	BBB targeting mechanism	Therapeutic molecule	Drug release mechanism	References
Biomimetic	Glioblastoma	C6 Glioma‐bearing mice	siRNA‐loaded CaP coated with HDL	ApoE	RMT	ATF5 siRNA	Release upon endosomal escape	Huang et al.^[^ ^159^ ^]^
Biomimetic	AD	AD mice	Quercetin‐loaded exosomes	Receptor‐mediated transcytosis		Quercetin	Release upon fusion	Qi et al.^[^ ^160^ ^]^
Biomimetic	PD	PD mice model induced with 6‐OHDS	hucMSC‐derived exosomes	Receptor‐mediated transcytosis		Growth factors, cytokines, and chemokines	Release upon fusion	Chen et al.^[^ ^161^ ^]^
Biomimetic	AD	SAMP8, SAMR1, and Sprague‐Dawley mice	ApoE3‐coated phospholipid carriers	ApoE3	RMT	ApoE3	Conjugated activity	Song et al.^[^ ^162^ ^]^
Biomimetic	Ischemic stroke	MCAO/R rats	Curcumin‐loaded exosomes	c(RGDyK) ligand	RMT	Curcumin	Release upon fusion	Tian et al.^[^ ^163^ ^]^
Biomimetic	Ischemic stroke	MCAO/R rats	Neutrophil membrane‐coated RCD NPs	SHp peptide	RMT	ß‐CD phenylboronic acid pinacol ester	ROS responsive	Dong et al.^[^ ^164^ ^]^
Biomimetic	TBI	Sprague‐Dawley rats (CCI)	NSC‐EVs	Receptor‐mediated transcytosis		Growth factors, cytokines, and chemokines	Release upon fusion	Sun et al.^[^ ^165^ ^]^
Biomimetic	TBI	Adult male Wistar rats	Poly(citrate‐gallic acid)‐exosome hybrid hydrogel	Local hydrogel injection		Human exfoliated deciduous teeth derived EVs	pH responsive	Li et al.^[^ ^167^ ^]^
Biomimetic	TBI	C57BL/6J male mice (CCI)	BV2‐derived EVs	RVG29 peptide	RMT	NR2B9c peptide	Release upon fusion	Haroon et al.^[^ ^168^ ^]^

### 3.1 Polymer NPs

Biodegradable polymer NPs (PNPs) have been studied extensively in cancer drug delivery and therapy. The biodegradability of PNP systems offers an ability for controlled and sustained drug release, often designed to degrade in the presence of biological stimuli. Various biodegradable PNP drug delivery systems include but are not limited to nanogel, poly (lactic*‐co*‐glycolic acid) (PLGA), poly (e‐caprolactone) (PCL), chitosan, hyaluronic acid, and dendrimers. Other poly ester NPs have been developed without the ability to degrade. However many of these systems have been shown to be retained in the body, leading to concerns of potential clinical efficacy. Therefore, the biodegradable PNP‐based systems are the main focus of this review. PNPs can be modified to obtain the desired characteristics for passive or active targeting when translocating across the BBB due to the variety of compositions and BBB targeting methods via conjugation chemistry.

#### 3.1.1 Glioblastoma

As discussed, the BBB is significantly altered in the context of glioblastoma progression, characterized by the formation of the BBTB. Due to this close integration of the tumor microenvironment and the BBB, it is exceedingly difficult to target tumor cells effectively. To this difficulty, the location of glioblastoma is notable for its proximity to healthy brain cells, complicating selective treatment. In 2019, Muniswamy et al. developed dendrimer‐cationized albumin (*d*CatAlb) via carboxyl‐modification on albumin.^[^ ^73^ ^]^ The prepared *d*CatAlb was adsorbed onto doxorubicin (DOX)‐loaded PLGA NP cores, constructing a novel hybrid DOX nanoformulation. The *d*CatAlb‐pDNP formulation showed a unique pH‐dependent DOX release profile, reduced hemolytic toxicity, higher drug uptake, and cytotoxicity in U87MG glioblastoma cells. Enhanced DOX delivery increased levels of the caspase‐3 gene in U87MG cells, indicative of anticancer activity through a caspase‐mediated apoptotic mechanism. The *d*CatAlb‐pDNP showed superior transepithelial permeation transport across a BBB epithelial model composed of bEnd.3 cells. The mechanism in which the *d*CatAlb‐pDNP permeates this model is considered AMT, based on the inherent ability of cationic serum albumin to enter the brain.^[^ ^74^ ^]^ Another mechanism of BBB transcytosis was demonstrated by Kou et al. utilizing L‐carnitine‐conjugated PLGA NPs (LC‐PLGA NPs).^[^ ^75^ ^]^ Investigators designed the PNP system to take advantage of the specific expression of Na^+^‐coupled carnitine transporter 2 (OCTN2) on both brain capillary endothelial cells and glioma cells. This interaction significantly enhanced the uptake of PLGA NPs in the BBB endothelial cell line hCMEC/D3 and the glioma cell line T98G. Additionally, in vivo mouse studies showed that LC‐PLGA‐NP highly accumulated in the brain, indicated by biodistribution and imaging assays. Compared to Taxol and paclitaxel‐loaded unmodified PLGA NPs, the L‐carnitine‐modified LC‐PLGA NPs enhanced antiglioma efficacy in both 2D‐cell and 3D‐spheroid models. This distinction indicates CMT for permeating the BBB, characterized by the presence of L‐carnitine in the nanoformulation. Finally, Chen et al. developed a Cyclo(RGD)‐decorated reduction‐responsive nanogel for mediated and targeted chemotherapy of integrin overexpressing glioblastoma cells in vivo.^[^ ^76^ ^]^ The nanoformulation was composed of cyclo(Arg‐Gly‐Asp) (cRGD) peptides complexed with DOX‐loaded poly(vinyl alcohol) nanogels (cRGD‐SS‐NGs). The presence of cRGD peptides on the surface of the particle allowed for RMT through the BBB, efficiently internalized by cells expressing *α*_v_ *β*_3_ integrin receptors. In contrast, nanoformulations without cRGD showed slow internalization and weak DOX fluorescence inside cells based on flow cytometry and fluorescence imaging done in studies performed comparing the targeted ligand.^[^ ^76^ ^]^ Furthermore, the researchers reproduced this distinction in vivo, as DOX‐loaded cRGD‐SS‐NG treatment significantly inhibited tumor growth, while DOX‐loaded SS‐NG treatment resulted in continuous tumor growth.^[^ ^76^ ^]^ Based on the studies described above, PNPs have shown great promise as nontoxic, bioavailable, and tunable nanotherapeutics for the treatment of glioblastoma in vivo, aided by their ability to utilize RMT, CMT, or AMT.

#### 3.1.2 Neurodegenerative Disease

Several recent studies have utilized PNPs to aid in the localization in the brain and target AD‐associated pathologies in neurons. To date, one of the most promising approaches for treating AD has been associated with the clearance of Aβ from the brain.^[^ ^77^ ^]^ In 2018, Carradori et al. designed an antibody‐functionalized PNP aimed to scavenge and clear Aβ from peripheral circulation and the brain. Biodegradable polyethylene glycol (PEG)‐conjugated NPs were surface functionalized with anti‐Aβ and were injected intravenously into AD‐like transgenic mice. The anti‐Aβ ligands on the surface of the NP led to a multivalent effect in binding to Aβ in both the brain and blood, which could then be cleared and excreted through the kidneys. Interestingly, investigators found that by reducing the amount of soluble Aβ in the peripheral blood using this nanotherapeutic, levels found in the brain were greatly diminished, attributed to the “sink effect” hypothesis.^[^ ^78^ ^]^ This discrepancy shifts the blood–BBB equilibrium of Aβ toward translocation out of the brain and into the peripheral blood, mediated by receptors such as lipoprotein receptor‐related protein 1 (LRP1) or p‐glycoprotein.^[^ ^79^ ^]^ In another study, PNPs were used for efficient drug delivery into the brain, crossing the BBB for the treatment of AD. Sanchez‐Lopez et al. designed PLGA‐PEG NPs loaded with memantine (MH), a drug approved for moderate‐to‐severe AD.^[^ ^80^ ^]^ In the past, MH has been inefficient for treatment due to its low solubility and inability to circulate effectively. MH is an uncompetitive N‐methyl‐d‐aspartate (NMDA) receptor antagonist, which preferentially binds to the NMDA receptor‐operated cation channels, overlapping the site of magnesium.^[^ ^81^ ^]^ As a result, MH decreases the excessive glutamate, which causes neuronal death in AD patients. Investigators were able to encapsulate MH into PLGA‐PEG NPs, which followed a sustained release profile, allowing a reduction in drug administration frequency in vivo from free drug solution. MEM‐PEG‐PLGA NPs could effectively cross the BBB with no disruption due to several characteristics of the NP. The spherical shape of the particle demonstrated an increase in transport across the BBB, aided by the grafting of PEG on the surface. The presence of PEG on the NP surface decreased the relative protein adsorption and, therefore, slowed down the clearance, improving circulation time. With an increase in circulation time, PEGylated NPs were able to accumulate more effectively in the brain.^[^ ^82^ ^]^ In a related study, Fornaguera et al. designed a PLGA NP synthesized by nanoemulsion templating using low‐energy methods as efficient nanocarriers (NCs) for drug delivery across the BBB in the context of neurodegenerative disease.^[^ ^83^ ^]^ Nanoemulsions are emulsions with droplet sizes, typically in the range of 20–200 nm, prepared using low‐energy emulsification methods. Of the various methods for low‐energy nanoemulsification, phase inversion nanoencapsulation methodology is highly advantageous for drug encapsulation, as it is performed at room temperature.^[^ ^84^ ^]^ In the case of polymer choice, PLGA was chosen for its biocompatibility and biodegradability in physiological conditions. Fornaguera and co‐workers loaded the drug loperamide into their PNPs, due to the inability of free loperamide to pass the BBB. Finally, the PNPs were functionalized with a monoclonal antibody against the transferrin receptor, widely expressed across cells of the BBB.

Although AD is the most commonly studied neurodegenerative disease due to its high prevalence and severity, avenues of treatment for HD have additionally been probed using nanotherapeutics. Strategies for treating HD include immunomodulation, modification or inactivation of HTT, inhibition of the NMDA extrasynaptic receptor, and silencing of the Huntingtin gene (*htt*). In 2021, Birolini et al. investigated a pathway that affects HD involving brain cholesterol. Peripheral cholesterol is unable to cross the BBB, and locally synthesized cholesterol is associated with synapse formation, maintenance and activity, and optimal neurotransmitter release.^[^ ^85^ ^]^ In the context of HD, brain cholesterol biosynthesis is reduced across several rodent models of HD.^[^ ^86^ ^]^ To efficiently deliver cholesterol through the BBB to treat HD, Birolini et al. developed a PGLA NP construct loaded with cholesterol and decorated with heptapeptide g7. Heptapeptide g7 has emerged as a brain‐targeting ligand, capable of transporting molecules into the CNS after systemic administration in rodents.^[^ ^87^ ^]^ By stimulating membrane curvature, g7 facilitates transcytosis through the BBB, leading to higher levels of localization in the brain.^[^ ^88^ ^]^ Investigators showed that the cholesterol‐loaded PLGA/g7 NPs (hybrid‐g7‐NPs‐chol) could rapidly reach the brain and target neural cells. With the sustained delivery of cholesterol to the brain utilizing the novel nanotherapeutic, endogenous cholesterol biosynthesis was enhanced, cognitive decline was mitigated, and motor defects were lessened in HD animals.

#### 3.1.3 Ischemic Stroke

**Figure 4:**
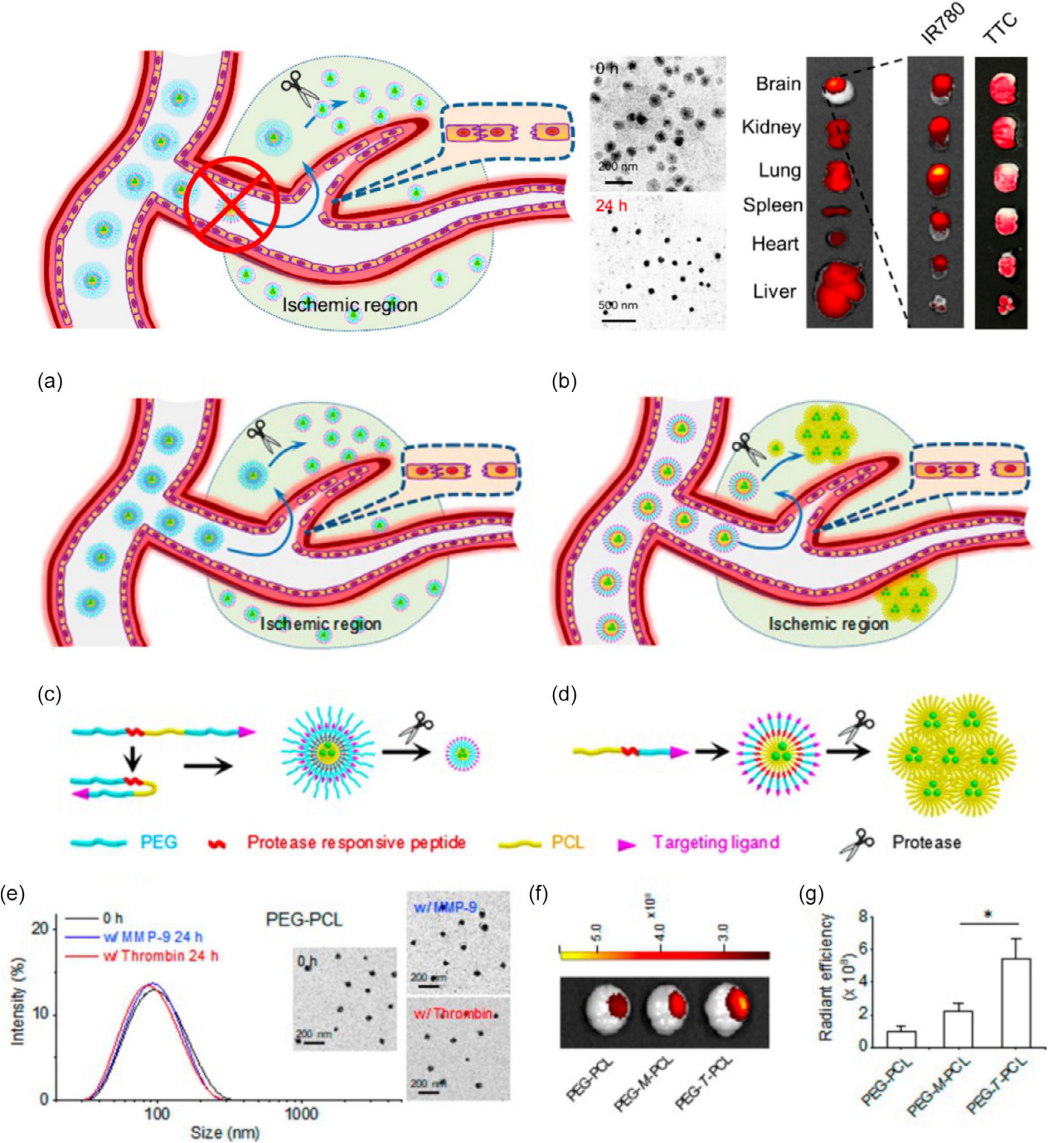
Overall schematic for the delivery and synthesis of a protease‐responsive and brain‐targeting polymer NP (top left). Transmission electron microscopy (TEM) images of the NPs at 0 and 24 h in the presence of a protease in addition to organ localization (top right). a,b) A schematic of the ischemic brain region for targeting by the NP system. c,d) The self‐assembly and protease‐mediated drug release for the NP therapeutic. e) Characterization of the NPs by TEM and dynamic light scattering and f,g) radiance efficiency is imaged and quantified. Reprinted (adapted) with permission.^[^ ^89^ ^]^ Copyright 2018, American Chemical Society.

Overcoming BBB transcytosis is crucial in the treatment of ischemic stroke, and advancements in nanotechnology have shown great promise for effectively targeting and delivering a therapeutic payload to the corresponding location. Furthermore, the oxygen‐deprived region of ischemia has been taken advantage of as a microenvironment for stimuli‐responsive drug release. In 2018, Guo et al. reported a strategy to synthesize protease‐responsive, brain‐targeting NPs for the improved treatment of ischemic stroke (**Figure** **4**).^[^ ^89^ ^]^ The polymeric matrix comprised PEG, PCL, and enzyme‐cleavable peptides. The peptides decorated on the surface, chlorotoxin (CTX), have high specificity and affinity for MMP‐2, often upregulated in the ischemic brain. Internalized in the particle was lexiscan (LEX), a small molecule utilized for myocardial perfusion imaging, and was additionally found to transiently enhance BBB permeability in mice.^[^ ^90^ ^]^ Following initial experimentation, investigators incorporated AMD3100, a well‐characterized antagonist of CXCR4, onto the surface of the particles. Additionally, LEX was replaced with the drug glyburide, a diabetes medication recently found to be effective for human stroke patients.^[^ ^91^ ^]^ Together, with a protease‐responsive payload release of glyburide, the formulated nanotherapeutic was able to treat and localize in the brains of mice injured by ischemia. In 2023, Zhang and co‐workers designed a mitochondrial‐targeted and ROS‐responsive polymeric NC to treat ischemic stroke via nose‐to‐brain administration.^[^ ^92^ ^]^ Utilizing a thioketal‐based ROS‐sensitive crosslinker, chitosan NPs were synthesized in the presence of puerarin (PU), which was loaded into the interior. The peptide SS‐31 was also modified on the chitosan surface to promote mitochondrial targeting. By taking advantage of an intranasal NP delivery route, these NPs were efficiently localized against the BBB, in which they could translocate through AMT. Although it is possible that the conjugation of SS‐31 would have aided in the transcytosis across the BBB, this possibility was not explored. Ultimately, they were delivered across the BBB of middle cerebral artery occlusion (MCAO) rats, and PU was released as a function of pathological ROS in the ischemia microenvironment. Specifically, the therapeutic restored mitochondrial function to MCAO rats and subsequently ameliorated neuroscore, decreased infarct volume, and alleviated brain edema in response to surgical MCAO injury.

#### 3.1.4 Traumatic Brain Injury

**Figure 5:**
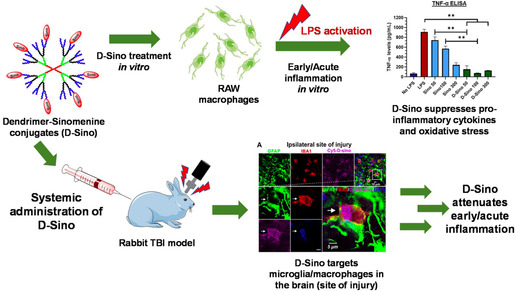
Schematic of polymeric dendrimer administration to both in vitro RAW macrophages and a TBI rabbit model. For in vitro experimentation, an ELISA for TNF‐α mRNA expression was conducted for various D‐sino conditions in addition to LPS and negative control conditions respectively. A) For in vivo experimentation, D‐sino was shown to target microglia and macrophages at the site of injury, resulting in attenuated early/acute inflammation. Reproduced and adapted under the Creative Commons Attribution (CC BY) license.^[^ ^93^ ^]^

Following TBI, neuroinflammation is orchestrated by activated microglia and macrophages at the injury site. These cellular polarizations play a critical role in the onset of various pathological events, such as BBB dysfunction, neuronal damage, and long‐term neuronal and behavioral deficits. Recently, Sharma et al. generated a polymeric dendrimer‐based therapeutic for the enhanced delivery of Sinomenine (Sino), a potent anti‐inflammatory and antioxidant drug as a potential therapy for attenuating early inflammation in TBI (**Figure** **5**).^[^ ^93^ ^]^ The dendrimer was synthesized using polyamino‐acrylate Michael addition strategies with hydroxyl functional terminal ends. To these hydroxyl groups, Sino was conjugated (D‐Sino). The D‐Sino dendrimer particles enhanced the intracellular ability of Sino due to rapid cellular uptake, significantly suppressed proinflammatory cytokines (tumor necrosis factor alpha (TNF‐a), IL‐1B, CCL‐3, and IL‐6), and reduced overall oxidative stress (iNOS and NO) in LPS‐activated murine macrophages (RAW 264.7) through the inhibition of the NF‐kB pathway. For in vivo studies, D‐Sino was administered to a rabbit model of pediatric TBI and specifically targeted activated microglia and macrophages at the site of injury. Additionally, D‐Sino was able to localize effectively in the brain, supporting the ability of the therapeutic to cross the BBB in vivo. Although PCL, PLGA, and chitosan‐based PNPs have largely dominated the field, dendrimers and dendrimer‐functionalized NPs have great potential in treating and attenuating brain‐associated diseases or injuries, such as TBI.

### 3.2 Lipid NPs

Characterized by their physical and chemical properties, lipid‐based NCs are categorized into niosomes, transferosomes, liposomes, solid lipid NPs (SLNs), and nanostructured lipid carriers (NLCs). Lipid NCs are attractive for their resistance to premature enzymatic degradation and subsequent enhancement in drug‐payload bioavailability. Furthermore, these NCs are efficient for diffusion through biological membranes. Of the categories of lipid‐based NCs, SLNs and NLCs remain the most useful for treating CNS diseases.^[^ ^94^ ^]^ SLNs were developed in 1990 and introduced to overcome disadvantages commonly associated with PNPs, liposomes, and nanoemulsions.^[^ ^95^ ^]^ Disadvantages include toxicity, instability, and low‐loading capacity with some formulations.^[^ ^96^ ^]^ SLNs are composed of solid lipids with bioactive compounds entrapped inside and stabilized by surfactants or polymers on the exterior. SLNs are biocompatible, show good stability and improved bioavailability of hydrophobic drugs, and can be tuned for sustained or burst drug release.^[^ ^97^ ^]^ NLCs have been developed more recently as a second generation of SLNs, formulated by incorporating both solid and liquid lipids. Although NLCs contain liquid lipids, they remain solid at room and bodily temperatures. NLCs improve upon SLNs regarding control release properties, improved encapsulation efficiency, and extended chemical stability.^[^ ^98^ ^]^

#### 3.2.1 Glioblastoma

**Figure 6:**
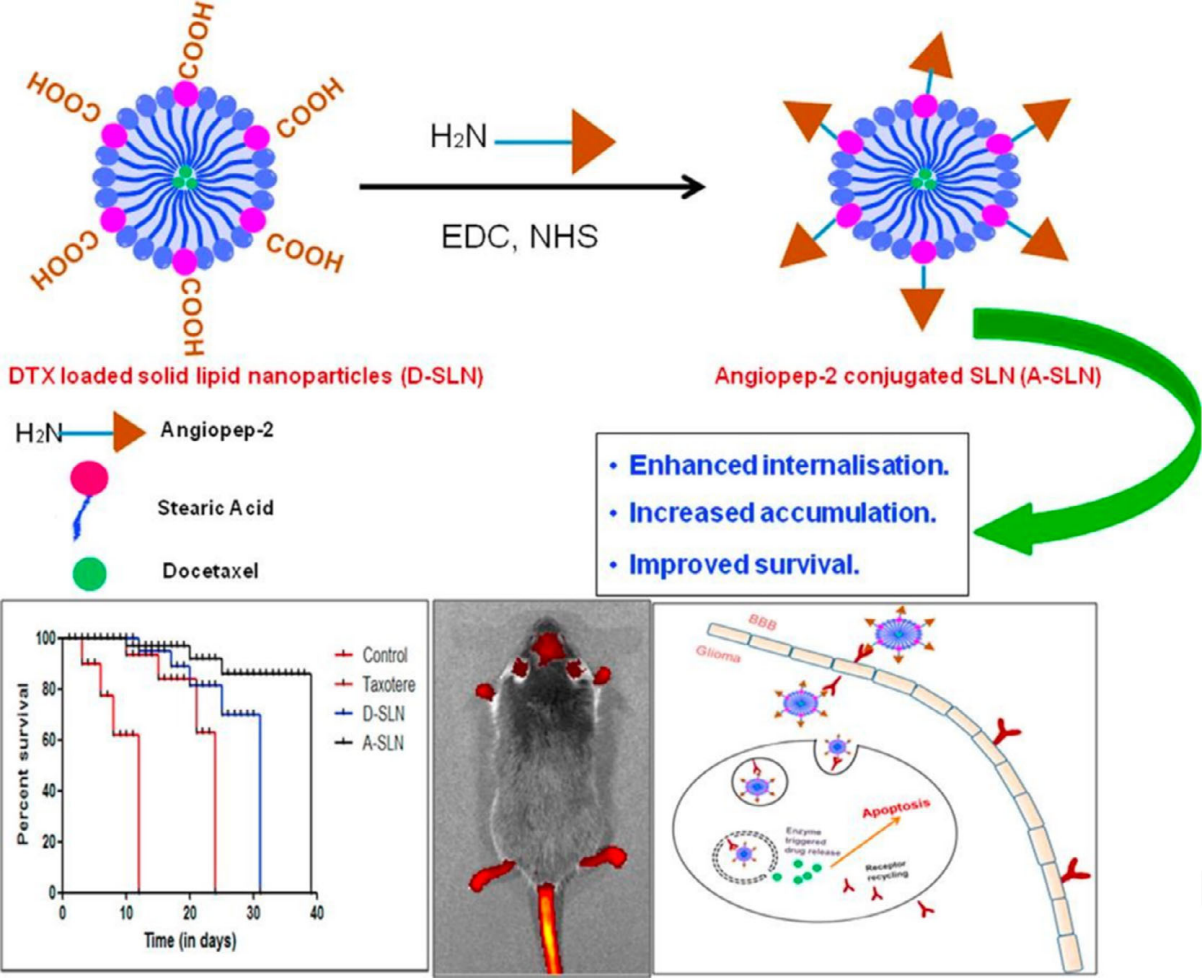
(Top) Composition schematic for the modification of docetaxel‐loaded SLNs with angiopep‐2. (Bottom) From left to right: Percent survivability of treated glioblastoma‐induced rats with corresponding conditions, local internalization of therapeutic, and schematic of BBB transcytosis and mechanism of action. Reproduced and adapted under the Creative Commons Attribution (CC BY) license.^[^ ^100^ ^]^

Due to their lipidic nature, SLNs and NLCs have some affinity for BBB transcytosis. This strategy takes advantages of both the RMT and AMT routes.^[^ ^99^ ^]^ Several studies have indicated the ability of angiopep‐2‐decorated particles to enhance drug delivery to glioblastoma multiforme cells due to its affinity toward LRP1 on the BBB. Kadari et al. showed this feature in 2018, by grafting angiopep‐2 onto the surface of SLNs encapsulating docetaxel (DTX) (A‐SLN) (**Figure** **6**).^[^ ^100^ ^]^ In vivo experiments in a glioblastoma‐induced C57BL/6 mouse model revealed considerable cellular internalization, cytotoxicity, and substantial apoptosis. In the absence of angiopep‐2, reduced cellular internalization and targeting were observed. Similar to the function of angiopep‐2, another study utilized melanotransferrin antibody (MA) to target the BBB and subsequent tumor cells. Melanotransferrin is a sialoglycoprotein expressed in the endothelial cells of the BBB and tumor cells. Kuo et al. utilized this interaction and developed SLNs conjugated with MA for the targeted etoposide (ETP) delivery across the BBB to glioblastoma cells.^[^ ^101^ ^]^ The results of the immunochemical staining demonstrated that MA‐conjugated ETP‐SLNs triggered the melanotransferrin‐mediated transcytosis and promoted the growth‐inhibitory efficacy to U87MG cells. Both of the previously discussed SLN systems used an RMT mechanism for BBB transcytosis, but AMT can also be utilized with SLNs and NLCs. Liu et al. demonstrated a dual‐prong approach to target the BBB by taking advantage of both AMT and RMT.^[^ ^102^ ^]^ Investigators prepared a cationic SLN (CSLN) decorated with OX26 Mab for the delivery of Baicalin across the BBB. OX26 was chosen for its affinity toward the transferrin receptor, commonly expressed on BBB endothelial cells. The OX26‐PEG‐CSLN possessed efficient entrapment efficiency of Baicalin and was superior in BBB transcytosis in relation to PEG‐CSLN and CSLN treatments, as shown by cerebrospinal fluid pharmacokinetic analysis using a rat model.

#### 3.2.2 Neurodegenerative Disease

Although strategies to treat AD, such as cholinesterase inhibitors and NMDA receptor antagonists, have shown promise, several factors limit their use in a clinical setting. Many of these drugs possess poor bioavailability, low solubility, poor permeability across cell membranes, and a high frequency of side effects, indicative that other treatments should be explored.^[^ ^103^ ^]^ Many alternative antioxidant and neuroprotective therapeutics have been reported to exhibit anti‐AD potentials, such as quercetin, curcumin, resveratrol, and ferulic acid.^[^ ^104^ ^]^ Recently, researchers have aimed to deliver such therapeutics to the brain with high bioavailability and stability for treating AD. For the delivery of both hydrophilic and lipophilic drugs with high stability, SLNs and NLCs remain the most efficient.^[^ ^105^ ^]^ In 2021, Saini et al. developed a ferulic acid‐encapsulated chitosan‐coated SLN to treat AD with a quality‐by‐design approach in mind.^[^ ^106^ ^]^ This approach taken by the investigators led to a thoroughly designed system with every parameter and clinical application in mind. SLN lipid composition was determined and optimized based on the ability to solubilize ferulic acid, increasing overall encapsulation efficiency to 74% after 12 h of incubation. Additionally, chitosan coating efficiency was optimized and found to be roughly 62%, values corresponding to those found in the literature. After chitosan coating, cSLNs were found to be 185 nm in diameter, with a zeta potential of 12.4 mV. Several techniques were utilized to characterize the cSLNs further, such as Fourier transform infrared spectroscopy, differential scanning calorimetry (DSC), power X‐ray diffraction, and field emission scanning electron micrscopy. Once the cSLNs were characterized extensively, the nanotherapeutic was tested in vitro and in vivo. Behavioral studies were conducted initially with AD‐induced rats via administration of streptozocin (STZ), in which cognitive ability was noticeably declined. In the case of cSLN administration to these AD‐induced rats, cognitive performance was significantly improved. Next, biochemical studies were conducted in each treatment group, quantifying levels of LPO, nitrate, protein, and acetylcholine esterase activity. All of these levels were increased significantly in STZ‐treated rat hippocampus and cortex brain regions, while glutathione (GSH) and superoxide dismutase (SOD) levels were diminished. Administration of cSLNs significantly attenuated oxidative stress parameters, most notably in chitosan‐coated SLN conditions where the route of administration was intranasal. These findings indicate that the newly developed nanotherapeutic can bypass the BBB and substantially improve ferulic acid delivery to the brain when administered intranasally. Finally, ferulic acid concentrations were analyzed in brain homogenates of animals treated intranasally with free‐ferulic acid, uncoated SLNs, and coated SLNs. Uncoated and coated SLNs were found in the homogenates at levels 5.42‐fold and 6.91‐fold, the levels found in free‐ferulic acid conditions. Surface coating on the SLNs produced remarkably higher levels, possibly due to extended nasal retention time, the opening of TJs of nasal epithelial cells, the positive charge of chitosan, and improved stability in the biological milieu of the developed nanotherapeutic. The successful outcomes provided by the quality‐by‐design‐mediated design of chitosan‐SLNs for the delivery of ferulic acid to the brain in AD rats show great promise for the expansion of therapeutics such as this in the application of other diseases.

#### 3.2.3 Ischemic Stroke

It is well known that modulating inflammation in the brain is a typical avenue for treatment in ischemic stroke, but many therapeutics aimed to ameliorate the pathology of the disease have poor circulation and diffusion in vascular systems due to hydrophobicity. To combat this issue, Wu et al. designed LNPs loaded with oleoylethanolamide (OEA), an endogenous highly hydrophobic molecule with outstanding neuroprotective effects.^[^ ^107^ ^]^ In past studies, Wu and co‐workers found that OEA could protect against ischemic injury by reducing inflammation. However, OEA has extremely poor water solubility, so the LNPs as a vehicle were developed. Researchers encapsulated the OEA in the LNPs with high encapsulation efficiency, facilitated by the lipidic nature of the particle itself. During synthesis, OEA was able to form hydrogen bonds with soybean lecithin (SPC), a component of the lipid layer of the particle. Additionally, the OEA could crystallize once inside the LNPs, increasing the drug loading efficiency by a factor of 4. The clever use of lipid‐based NPs with such a hydrophobic drug allowed for the enhanced drug loading efficiency to be observed, subsequently increasing the bioavailability of OEA at the site of delivery. One of the advantages of the LNPs used in this study was the ability for sustained drug release with high drug loading. In drug release experiments, the LNPs showed a small burst release within the first 8 h, followed by a controlled, sustained release over the next 40 h. The crystallized nature of OEA in the center of the LNPs allowed for the slow diffusion observed, which is uncommon in many LNP‐based systems. Systematic experiments were performed on rats to assess the in vivo neuroprotective effects of the LNPs on ischemic cerebral injury. Specifically, the ischemic penumbra was analyzed for its visualization and recovery from OEA‐LNP administration, through positron emission tomography (PET) of [^18^F] FDG, employed to assess cerebral glucose metabolism. All PET data was collected 2 h after reperfusion to eliminate the effect of inflammation. Interestingly, roughly 41.7% of the right cerebral hemisphere from rats administered with OEA‐LNPs exhibited significantly higher signals compared with normal tissues. After 2 h, this enhancement was increased to 83.6% of the right cerebral hemisphere. Additionally, the treatment of free OEA versus OEA‐LNPs displayed a significant difference in cerebral infarct volume, of 330 ± 26.9 and 78 ± 18.4 mm^3^, respectively. The data reflects the ability of the LNPs to efficiently deliver OEA to ischemic regions of the brain in rats, resulting in much less brain damage than rats in opposing groups.

#### 3.2.4 Traumatic Brain Injury

As discussed, in the context of TBI, pathological alterations to the BBB structure result in leakiness inside and around the cortical lesion, as well as in the hippocampus and hypothalamus. The damaged state of the BBB has been investigated for potential pathways of entry to the brain for TBI therapy. In 2022, Khalin et al. designed lipid nanoemulsion droplets (LNDs) loaded with a fluorescent dye and investigated the biodistribution of both 30 and 80 nm particles in a mouse brain following TBI.^[^ ^108^ ^]^ The fluorescent LNDs were formulated using nanoemulsification strategies, where oil, surfactant, and dye are premixed together, followed by the addition of water. After 90 min of injection into the femoral artery of TBI‐associated mice, both 30 and 80 nm LNDs were observed to localize with the compromised BBB in the cortex and the hippocampus, suggesting specific accumulation in injured tissue. Using higher‐magnification imaging, researchers found that the LNDs were incorporated into microvascular clots or vascular occlusions (VOs). VOs are present in the histopathological analysis of the TBI brain, but little is known about the kinetics of their formation and their role in the pathophysiology of TBI.^[^ ^109^ ^]^ Interestingly, it was observed that 30 nm LNDs tended to extravasate out of VOs and into the brain parenchyma more readily than 80 nm particles. This finding directly indicated the increased permeability of the BBB at VO sites, specifically allowing 30 nm LNDs transcytosis in TBI‐associated brains in vivo. It was ultimately concluded that LNDs do not cross the healthy BBB, but penetrate the BBB at the sites of VOs, suggesting a role of VOs in BBB permeability. In another study, a TBI‐targeted lipid‐covered radical scavenger NP was developed to deliver nimodipine (Np) (CL‐PPS/Np) in order to inhibit Ca^2+^ influx in neurons by Np and to scavenge ROS in the brain trauma microenvironment by poly(propylene sulfide)_60_ (PPS_60_), preventing TBI‐associated secondary injury.^[^ ^110^ ^]^ Researchers used a short peptide CAQK to target the TBI area of the brain based on its capacity to interact with the proteoglycan complex, which is commonly elevated in TBI‐associated brain regions. With CAQK conjugated to the surface of the CL‐PPS/Np, the therapeutic was found to highly accumulate in the brain of mice following tail injection after 6 and 24 h, analyzed using a de vivo imaging system with NIR dye. Additionally, CL‐PPs/Np was aimed to reduce the breakdown of the BBB, and subsequent edema, through PPS_60_ ROS scavenging. After injection of CL‐PPS/Np, brain edema was assessed using magnetic resonance imaging (MRI) 3 days after TBI. When compared to untreated TBI and free Np‐treated, reduced water content in the brain was observed for CL‐PPS/Np conditions. On the other hand, hypertensity volume was also reduced for CL‐PPS/Np compared with CL‐PLGA/Np‐treated and CL‐PPS‐treated groups. Finally, calcium overload, often leading to neuronal apoptosis, mitochondrial dysfunction, and increased ROS production, was limited through the administration of CL‐PPS/Np. To investigate intracellular Ca^2+^ influx, a single‐cell suspension of mouse brain tissue was made, and Ca^2+^ was detected using a Fluo‐3AM calcium probe. Treatment with CL‐PPS/Np was observed to significantly decrease the levels of Ca^2+^ influx in comparison to untreated TBI, free Np, CL‐PPS, and CL‐PLGA/Np groups. Both studies discussed that lipid‐based NPs could penetrate the BBB through AMT and RMT pathways, respectively. More importantly, the lipid‐based NPs utilized had increased circulation time for hydrophobic payloads, enhancing the delivery into TBI areas of the brain.

### 3.3 Gold Nanomaterials

Various gold nanomaterials, including NPs, nanorods, and nanoclusters, have demonstrated the ability to cross the BBB and enter the brain. Gold is innately an inert material, so the biocompatibility of gold‐based nanomaterials is highly dependent on the surface ligands capping the particle, cluster, or rod. The therapeutic properties of gold nanomaterials highly depend on their size, shape, and surface ligands capping the surface. The surface‐capping ligands provide a diverse range of potential therapeutic applications. Gold nanomaterials also have unique optical properties that can be advantageous for sensing in vivo. Gold nanoclusters are less than 5 nm in size and can have varying fluorescent emissions depending on the ligand and oxidation state of the gold. The bandgap size between the highest occupied molecular orbital and the lowest unoccupied molecular orbital is critical in the catalytic properties of gold nanoclusters. Gold NPs and gold nanorods have surface‐enhanced Raman spectroscopy, which gives a large Raman scattering enhancement of molecules capping the surface of the particles and is of interest for imaging and biosensing in vivo. They have also been utilized in combination with other nanomaterials for targeted release and multifunctional properties.^[^ ^111^ ^]^

#### 3.3.1 Glioblastoma

**Figure 7:**
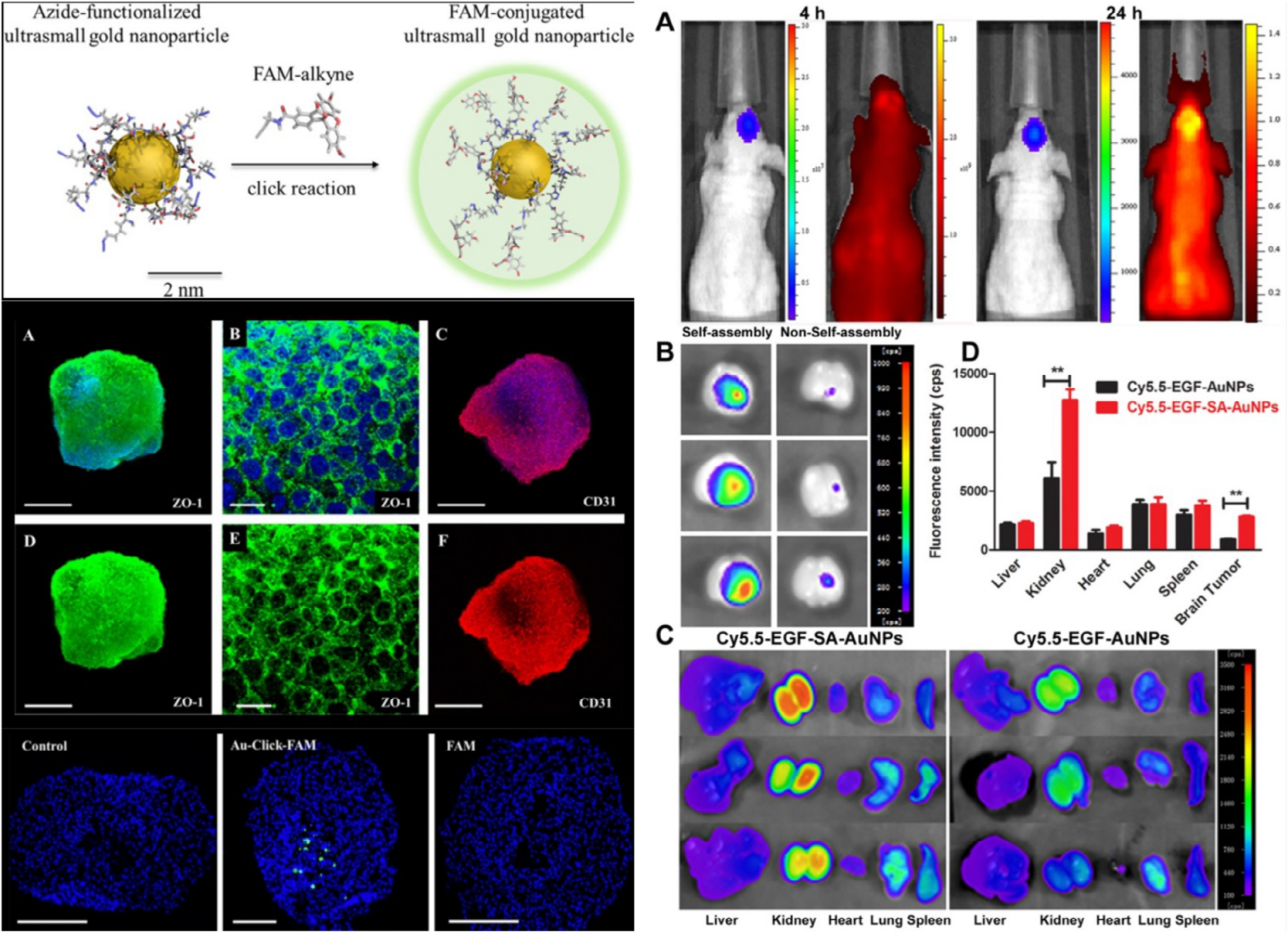
Top left: Synthetic schematic for the azide‐click reaction for FAM‐conjugated ultrasmall gold NPs. Bottom left: A–F) Fluorescence microscopy images of brain tissue slices showing localization of the particles. Reproduced and adapted under the Creative Commons Attribution (CC BY) license.^[^ ^119^ ^]^ Right: A–D) The in vivo localization of the NPs in the various organs, highlighting the specificity for the brain‐tumor region. Reproduced and adapted under the Creative Commons Attribution (CC BY‐NC) license.^[^ ^115^ ^]^

In 2012, Etame investigated the effect of PEG on Au NPs.^[^ ^112^ ^]^ In this study, AuNPs were synthesized using the method proposed by Frens, which is based on reduced ionic gold in AuNP fabrication protocol.^[^ ^113^ ^]^ Folic acid and BSA‐covered gold nanoclusters were shown to pass the BBB in a mouse model and enhance accumulation in glioma tumors for enhanced radiation efficiency and decreased exposure time.^[^ ^114^ ^]^ The accumulation was highest in the brain tumor, followed by the kidney, liver, spleen, lung, and then the general brain. This study shows the potential of targeting brain tumors and achieving BBB passage to enhance the efficacy of radiation therapy. Due to the ability of AuNPs to clear the body quickly, Feng et al. developed gold NPs that were crosslinked with dithiol‐polyethylene glycol to form a nanosphere of about 80 nm in size and functionalized with epidermal growth factor peptide to target tumors in the brain utilizing the disruption that occurs during formation of the BBTB (**Figure** **7**).^[^ ^115^ ^]^ The NPs were capped with a pH‐sensitive linker conjugated to doxorubicin for targeted drug release in the acidic environment in tumor conditions. Utilizing the changed physiology of the BBB during brain tumors, these large NP aggregates can be delivered through the BBB and release the drug to the brain tumor. To confirm delivery across the BBB, an in vivo mouse model was used, and the treatment was injected into the tail. The contents of AuNPs in the brain were measured with inductively coupled plasma–mass spectrometry. The circulation half life of the NP assemblies was almost 12 h, while the individual NPs only had a half‐life of 5 h. Both the assemblies and individual NPs had similar rates of accumulation in the normal brain with a gold content of .68% and .51% ID g^−1^, respectively. The accumulation of the assemblies in the brain tumor was 3.7 times that of the individual AuNP, with gold contents of 5.6% and 1.5% ID g^−1^, meaning it has much better accumulation in the tumor and can effectively target the brain tumor.

#### 3.3.2 Neurodegenerative Disease

Ruff et al. conducted an important study looking at the effects of gold NP and nanorod size, charge, shape, and concentration when bound to β‐amyloid‐specific peptides on BBB integrity.^[^ ^116^ ^]^ They compared NPs and nanorods that would bind selectively with β‐amyloid with sphere sizes ranging from 1.4 to 45 nm and rod sizes with a length between 40 and 60 nm and a width of 11–15 nm. The authors only saw disruption of BBB integrity with the 1.4 nm gold NPs, which they determined by checking transendothelial electrical resistance (TEER) measurement and the capacitance of the cell layer. With 1.4 nm NPs, they saw an increase in capacitance and a decrease in the TEER measurements. They could confirm that a negative charge inhibited gold NPs and nanorods ability to pass the BBB. Still, PEG coating could help stabilize the NPs and increase the ability to pass the BBB. While this study showed BBB disruption with ultrasmall nanoclusters, many groups have reported no BBB disruption with the use of these particles. Ultrasmall dihydrolipoic acid gold nanoclusters and histidine gold nanoclusters, about 3.4 nm in size, were used to modulate the fibrillation of alpha‐synuclein to mitigate PD pathology.^[^ ^117^ ^]^ Both gold nanoclusters were shown to be able to cross the BBB in vivo, and the accumulation of the histidine gold nanocluster in the brain was able to be investigated based only on its inherent fluorescence features. The similar emissions of the histidine gold nanoclusters and the mouse brain homogenate after treatment confirmed the uptake of the particles into the brain. As the nanoclusters were not designed to target any active transport pathways into the brain, the ability to diffuse through TJs was confirmed. In 2022, Ge et al. created gold nanorods where the ends were coated with ceria NPs (CeO_2_) for photocatalytic elimination of ROS and photothermal therapy of Alzheimer's under NIR light.^[^ ^118^ ^]^ The primary goal of photothermal therapy was to eliminate Aβ from the brain. To improve treatment efficacy, KLVFF, a peptide that inhibits Aβ, was conjugated to the center of the NP through an absorption‐mediated method. They tested the efficacy of this nanosystem in passing the BBB through an in vitro transwell model utilizing bEnd.3 endothelial cells, as well as in vivo. This stimuli‐responsive gold nanosystem was also shown to increase the permeability of the BBB in vivo as under NIR light, there was a much higher accumulation of theNP in the brain, which led to the effectiveness of a photothermal approach to BBB passage, although the NP could still enter the brain at lower concentrations after a longer time without the use of NIR light.

#### 3.3.3 Ischemic Stroke

In 2020, Sokolova et al. tested the passage of ultrasmall gold NPs with a diameter of 2.3 nm into a spheroid brain model utilizing six cell types that formed an in vitro BBB (Figure 7).^[^ ^119^ ^]^ Earlier, these NPs were shown to penetrate cell nuclei in a simpler 3D brain spheroid model as well.^[^ ^120^ ^]^ They tested the delivery of the NPs past the BBB under normal and hypoxic conditions.^[^ ^119^ ^]^ First, these particles were shown to penetrate cell nuclei in models, including astrocytes and pericytes surrounded by endothelial cells, to look at the ability of these particles to enter cell nuclei. Then, to investigate the ability of the NPs to cross the BBB, an in vitro 3D BBB model including astrocytes, pericytes, endothelial cells, microglia cells, oligodendrocytes, and neurons was generated, which regulated the passage of substances beyond the in vitro barrier. To induce hypoxic conditions seen in ischemic stroke and replicate the BBB damage that occurs, the spheroids were put in a system with 99.9% N_2_ and 0.01% O_2_ for 24 h before NP delivery. In ischemic conditions, the BBB permeability increased as measured by dyes, and the uptake of gold NPs into the spheroids increased as well. The gold NP uptake was caused by the concentration gradient between the NPs outside of the spheroid and its interior, but the dye could not diffuse through the BBB. This displayed that the BBB had normal functionality without ischemic conditions, and the NPs had a separate mechanism of entry that the dye could not undergo. After only a few minutes, the NPs were found almost exclusively in the inner cells of the spheroid, with almost none in the endothelial cells or pericytes, indicating that direct diffusion was most likely the mode of transport because endocytosis, exocytosis, and transcytosis cannot occur on that short of a timeframe. NP delivery across the in vitro BBB was enhanced under ischemic conditions. Nazarian et al. co‐delivered modafinil‐coated AuNPs with mesenchymal stem cells (MSCs) to treat ischemic stroke.^[^ ^121^ ^]^ Modafinil has been shown to reduce inflammation and oxidative stress and increase hippocampal neuron proliferation and differentiation. MSCs can differentiate into neurons and have been used for therapeutic use in the past to treat ischemic stroke. The AuNPs ability to pass the BBB increased the efficacy of modafinil, although the exact mechanism of passage was not analyzed in this study.

#### 3.3.4 Traumatic Brain Injury

Inflammation is a major factor in TBI and the polarization of macrophages and microglia from the proinflammatory M1 state to the anti‐inflammatory M2 state is an important potential therapeutic for the treatment of these diseases. Lin et al. showed the ability of dihydrolipoic acid gold nanoclusters to polarize macrophages from proinflammatory to anti‐inflammatory states (M1→M2) and enhance their polarization by modifying the cluster with zinc.^[^ ^122^ ^]^ These nanoclusters could reduce neuron ROS‐induced apoptosis as well as inflammation in vivo, showing the ability of the NP to reach the injury site postinjection. More recently, Liu et al. designed and developed Au‐based clusterzymes, Au_24_Cu_1_ and Au_24_Cd_1_, with inherent high catalytic activity and favorable enzymatic selectivity, superior to natural antioxidants.^[^ ^123^ ^]^ The gold clusterzymes displayed characteristic CAT and SOD‐like activities, efficiently able to remove several types of ROS from the site of injury. Using a TBI model, in vivo results showed the accumulation of the clusterzymes at the site of injury, where they were effective in reducing oxidative stress and suppressing neuroinflammation. This reduction of oxidative stress contributed to the recovery of TBI‐induced weight loss and improvements in spatial memory and learning ability post‐TBI. Additionally, the ROS scavenging properties of the therapeutic were able to reduce the overall infarct volume, improving cognitive and motor deficits after TBI. In another recent work, Hunt et al. showed the potential of gold NPs for a protective effect on the BBB.^[^ ^124^ ^]^ This work showed that 20 nm citrate‐capped AuNP delivery in vivo led to a decrease in BBB permeability as tested through transvascular filtration per surface area. There were also key changes to cerebrovascular function. Overall the AuNPs may be able to help increase BBB integrity through interactions with endothelial cells although these interactions should be further studied to determine potential detrimental off‐target effects such as arterial stiffening. Although this work was not testing the effects on a TBI model, targeting an increase in BBB integrity is an important target for the treatment of TBI that should not be overlooked.

### 3.4 Magnetic and Inorganic NPs

Among various NP systems, MNPs have since emerged as multifunctional NPs with unique tunable properties under external stimuli, such as magnetic fields and electric magnetic fields. In research, MNPs have become attractive candidates for drug and gene delivery applications, medical imaging, and hyperthermia induction in cancer treatment. Due to their magnetic influence, MNPs can be manipulated remotely once internalized to direct and promote localization in the desired body region. Most times, this can increase the efficacy and selectivity of the treatment, subsequently mitigating off‐target effects and increasing payload bioavailability at the site of interest. In the context of medical imaging, MNPs, typically iron oxide NPs (IONPs), are used to reduce the MRI signal intensities on T_2_‐ and T_2_*‐weighted images, resulting in magnetic field inhomogeneity.^[^ ^125^ ^]^ Hyperthermia, which utilizes heat generation of MNPs for the thermal treatment of cancer, remains an attractive and noninvasive approach. MNPs can be synthesized using various strategies, such as aqueous coprecipitation and thermal decomposition, among others.^[^ ^126^ ^]^

#### 3.4.1 Glioblastoma

Due to the complex pathology associated with glioblastoma, brain tumor‐targeted delivery of diagnostic contrast agents has remained an elusive goal. Despite these challenges, researchers have aimed to design multimodal systems with numerous approaches for payload delivery and tumor localization for the treatment of glioblastoma. In 2020, Duan et al. designed a complex, multifaceted NP system for targeted delivery into the brain tumor tissues.^[^ ^127^ ^]^ The NP design consisted of an IONP core precipitation with a conjugated polymer for photoacoustic imaging (PAI) and PLGA, resulting in a 64 nm magnetic‐polymer core (CPIO). The core of the particle enables multimodal imaging both in vitro and in vivo, combining MRI with PAI techniques. The CPIO NPs synthesized were subsequently encapsulated into cRGD‐labeled brain tumor cell membranes (CM) as “tactical shells” to improve brain targeting.^[^ ^128^ ^]^ Duan et al. could decorate brain tumor cell membranes with cRGD groups using azide‐bioorthogonal click chemistry (CD). To encapsulate CPIO in the cRGD‐decorated CMs, CPIO NPs were extruded against cRGD‐CMs to yield cRGD‐CM‐CPIO NPs. For in vitro experiments, cRGD‐CM‐CPIO displayed negligible toxicity against both brain tumor C6 cells and fibroblast NIH‐3T3 cells, up to 120 μg mL^−1^. Interestingly, regarding bare CPIO NPs, CM‐CPIO and cRGD‐CM‐CPIO both showed significantly higher cellular uptake, indicating the homotypic targeting role of the cell membrane coating strategy, which is independent of the RMT pathway. As follows, brain tumor‐targeting efficiency in animal models was investigated using a brain tumor‐bearing BALB/c nude mice model. MRI was conducted to investigate the ability of cRGD‐CM‐CPIO to localize in brain tumor tissue. After 12 h post‐injection, the T_2_‐weighted MRI signal was the darkest for cRGD‐CM‐CPIO when compared to control treatments, suggesting higher accumulation in tumor brain tissue. The MRI data were then crossvalidated using PAI, which also indicated tumor brain tissue localization by cRGD‐CM‐CPIO. To this end, the CPIO NPs maintained high brain localization and subsequent bioavailability due to the multimodal spatial control imparted by the MNPs but also RMT provided by the cRGD peptides.

#### 3.4.2 Neurodegenerative Disease

**Figure 8:**
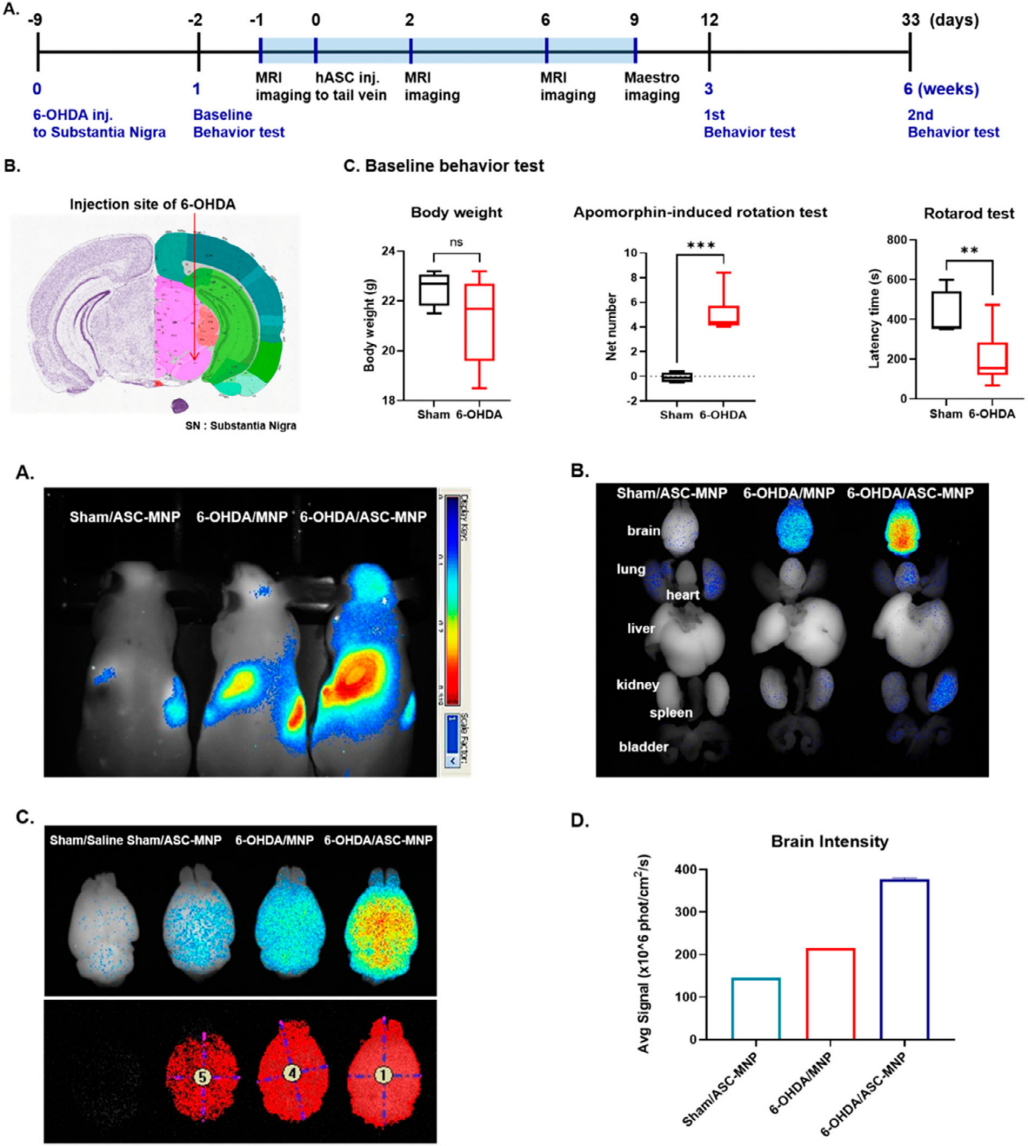
Experimental design, in vivo behavioral testing, and localization of a core–shell IONP‐PLGA NP therapeutic in mice. A (Top): Experimental design and timeline of in vivo experimentation. B (Top): Injection site of the therapeutic. C (Top): Behavioral testing for treated mice with respect to sham conditions. A–D (Bottom): Localization and retention of the NPs for each organ, respectively. Enhanced brain imaging and quantification were used for more specific localization within the brain tissue. Reproduced and adapted under the Creative Commons Attribution (CC BY) license.^[^ ^129^ ^]^

In 2021, Kim et al. aimed to deliver MNP‐labeled human adipose‐derived stem cells (hADCS) to the brain of a 6‐hydroxydopamine (6‐OHDA)‐induced PD mouse model (**Figure** **8**).^[^ ^129^ ^]^ To label the hADCSs, 50‐nm NIR dye‐labeled silica‐coated MNPs were obtained from a supplier and incubated with the stem cells in vitro before washing and collecting the cells. Investigators administered the MNP‐labeled hASCs via tail vein injection, before analyzing the mice over the following 33 days. After 12 days of hASC transplantation, appreciable motor recovery was observed in the PD model, signified by apomorphin‐induced rotation tests and rotarod tests. Further, in vivo MRI and fluorescence imaging of the MNPs and NIR dye‐labeled hACSs were obtained to verify an efficient transplantation method. Both imaging techniques showed the targeted distribution of hASCs in the brain of 6‐OHDA‐induced PD mice, and over the course of 33 days, they showed a significant recovery of motor function. Finally, behavioral tests confirmed the recovery of nigrostriatal dopamine neurons by hACS transplantation in the 6‐OHDA/ASC‐MNP mice. Although dopaminergic neuronal cell death was observed sharply following 6‐OHDA injection, recovery was observed with MNP‐assisted hASC transplantation.

Recent advancements in nanotechnology have improved therapeutics and diagnostic agents for treating and detecting early biomarkers of AD, respectively. In some cases, researchers have aimed to both diagnose and treat AD pathology utilizing complex nanotherapeutics. In 2015, a MNP‐based NP system was designed by Hu et al. for the labeling of Aβ plaques in vivo for MRI detection techniques, while employing a “smart” H_2_O_2_‐responsive system for the delivery of rutin, a therapeutic agent.^[^ ^130^ ^]^ In this system, 13 nm oleic acid‐capped MNPs were synthesized and coated using a mixture of DSPE‐PEG‐Congo Red and DSPE‐PEG‐phenylboronic acid. The lipidic nature of DSPE allowed the ligands to associate with the oleic acid‐capped groups of the particle by hydrophobic effect, leaving the congo red and phenylboronic acid groups to face the exterior. Once coated, rutin was then incorporated into the surface, primarily binding with phenylboronic acid groups via the vicinal diols on the flavonoid structure. For in vivo analysis, the MNP‐based system was administered to AD‐transgenic mice via tail vain injection, followed by MRI and immunohistochemical analysis. After injection, MRI images of AD mice brains were taken to assess the detection and labeling enhancement of Aβ provided by the MNP. MRI images resulted in detecting many dark spots in AD mice brains using in vivo T_2_*‐weight MRI. Finally, Nissl staining was performed to stain Aβ deposition and nerve cells in the brain. In conditions treated with congo red/rutin‐MNPs, there was a significant decrease in Aβ plaque loads. Interestingly, congo red‐MNPs and rutin‐MNPs, respectively, did not elicit therapeutic effects close to that of the combination due to the inability of congo red to scavenge ROS and the inability of rutin to target the pathological plaques effectively.

#### 3.4.3 Ischemic Stroke

**Figure 9:**
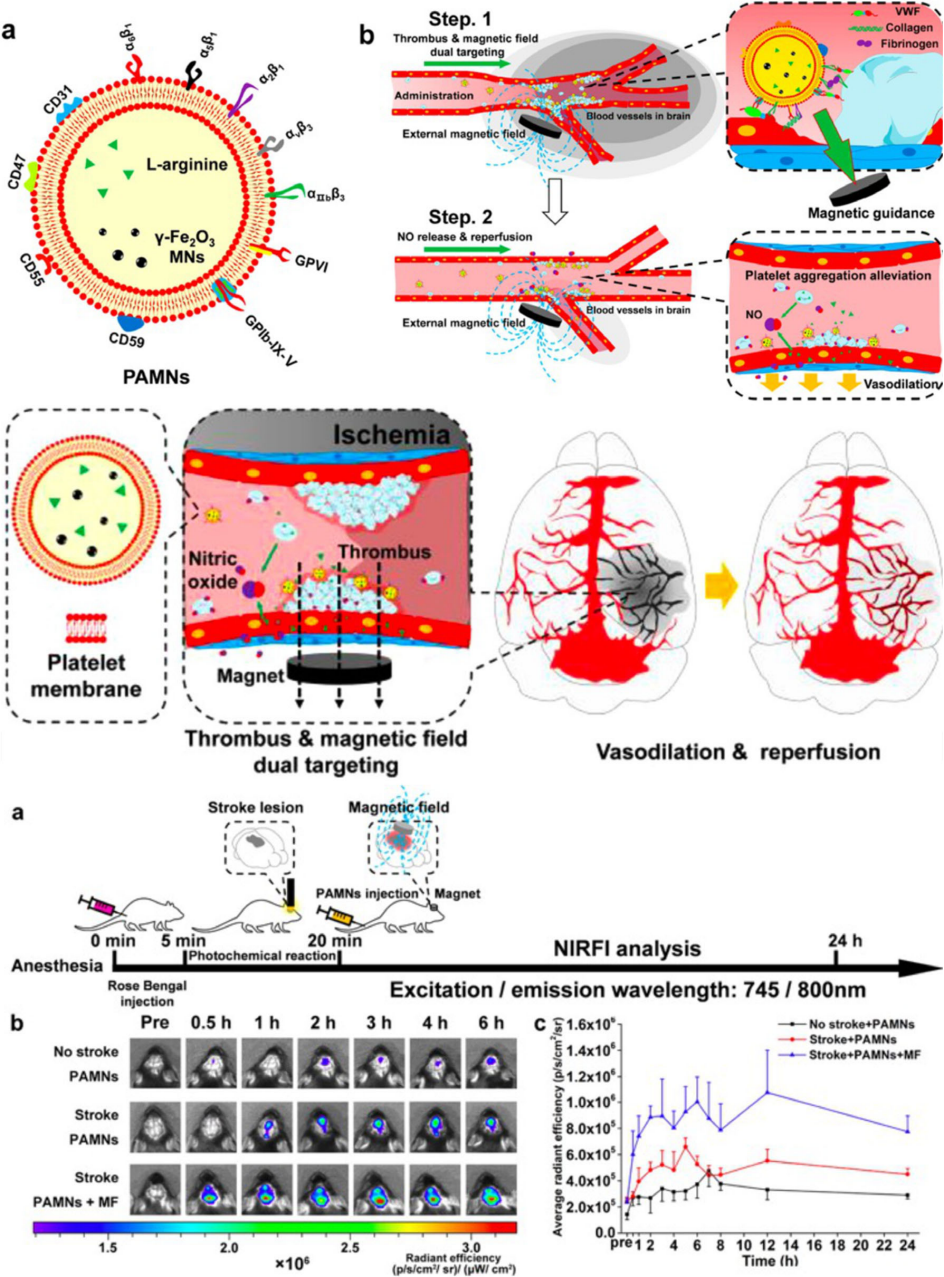
Overview schematic for the delivery and localization of a biomimetic nanocarrier comprising a PLT membrane, loaded with L‐arginine and Fe_2_O_3_ NPs in mice. a,b) (left) A schematic for the delivery of the therapeutic into the ischemic legion via magnet‐assisted targeting. Additionally, the endogenous production of NO from L‐arginine is displayed along with the mechanism of action. a–c (right)) In vivo experimental design and timeline, along with localization of the therapeutic in the brain over 6 h. Reprinted (adapted) with permission.^[^ ^134^ ^]^ Copyright 2020, American Chemical Society.

Thrombotic vascular diseases, such as ischemic stroke, are often difficult to treat and characterized by the narrow therapeutic time window (4.5 h) available.^[^ ^131^ ^]^ Many approaches in treating ischemic stroke have revolved around prolonging this therapeutic window, resulting in the least amount of brain tissue injured. Nitric oxide (NO) is an important signal‐modulating molecule for maintaining vascular homeostasis, regulating vasodilation, and inhibiting platelet (PLT) activation and aggregation.^[^ ^132^ ^]^ The delivery of NO in NP‐based carriers has proved difficult and inefficient, due to rapid quenching by molecular oxygen.^[^ ^133^ ^]^ To ameliorate this issue, NO donors such as L‐arginine can be used as biosynthetic precursors to endogenously produce NO in the brain. To test this strategy, in 2021, Li et al. fabricated a biomimetic NC consisting of PLT membrane envelope loaded with L‐arginine and Fe_2_O_3_ MNPs (PAMNs) for thrombus‐targeted delivery of L‐arginine and in situ generation of NO (**Figure** **9**).^[^ ^134^ ^]^ Li and co‐workers synthesized the PAMNs by extrusion techniques, in which the acquired PMVs and MNPs were mixed with L‐arginine. Following, the mixture was extruded through porous membranes for homogenous, monodispersed populations of PAMNs. For in vivo experiments, PAMNs were administered to mice with ischemic injury, followed by MRI and NIR fluorescence imaging strategies. PAMNs were initially labeled with DiR, a near‐infrared fluorescent dye. After injection, a signal increase was observed in the ischemic lesions of stroke‐bearing mice, indicating PAMN adherence to sites of vascular injury. Applying a magnetic field above the lesion area resulted in a significant and rapid signal rise in the same region. These results were indicative of magnetic field‐assisted enhancement of PAMN accumulation in the ischemic lesion 6 h after injection. To assess the efficiency of NO production via delivered L‐arginine, Li et al. used a full‐field laser perfusion imager to quantitatively detect the recanalization of blood vessels in ischemic lesions. Compared to control groups injected with saline and experimental groups injected with free L‐arginine, the ischemic area showed significant recanalization at 0.5–1 h post‐PAMN administration. Researchers recorded that blood vessels with dynamic blood flow reappeared, and revascularization rates and blood flow were further enhanced after applying the external magnetic field gradient.

#### 3.4.4 Traumatic Brain Injury

Magnetic particle imaging (MPI) is a promising new technique that utilizes internalized MNPs as contrast agents for higher sensitivity and fast image acquisition, among other advantages.^[^ ^135^ ^]^ Taking advantage of this strategy, in 2014, Das et al. used chitosan and polyethyleneimine (PEI)‐coated magnetic micelle (CPMM) designed by Wang et al. as a potential MRI contrast agent and nonviral vector for intranasal mild TBI (mTBI) diagnosis.^[^ ^136^ ^]^ The system was composed of an oleic acid‐capped Fe_2_O_3_ NP, encapsulated in a polymeric micelle comprising mPEG and PLA. The resulting micelle was then coated with chitosan and subsequently PEI before purification. The reporter plasmid, pCMV‐tdTomato, was complexed onto the surface of the particle by electrostatic interaction. For in vivo treatment, mTBI rat model was chosen for cellular uptake and brain localization. Upon administration, rats were subjected to a magnetic field for enhanced CPMM brain entry. Compared to groups not exposed to a magnetic field, significantly higher CPMM localization was observed in regions associated with TBI, as suggested by colocalized Fluoro‐Jade B and Prussian blue staining. Additionally, RFP transfection was observed in both the cortex and hippocampus, regions of the brain most commonly associated with mTBI‐mediated neurodegeneration.^[^ ^137^ ^]^ Further, an evans blue extravasation study was conducted to investigate the pathway in which the CPMMs penetrate the BBB. Evans blue entry to the brain was observed in rats that were exposed to mTBI, but not in sham groups. Likewise, upon CPMM administration, colocalization signals of the NPs and evans blue were observed, suggesting that particle transcytosis into the brain parenchyma was due to compromised cerebral microvasculature. Ultimately, the CPMMs were found to be nontoxic in vivo and were successful in localizing in the brain of mTBI model for gene transfection and potential early diagnosis. Cerium oxide NPs (CeNPs) are potent ROS scavengers characterized by the self‐regenerative redox cycling of Ce^3+^ and Ce^4+^ on their surface. Additionally, unlike most small‐molecule antioxidants, CeNPs possess multienzymatic activities for several different types of ROS. In 2022, Kang et al. utilized novel biocompatible polymer‐coated ultrasmall CeNPs (CX201) for BBB transcytosis and subsequent oxidative stress attenuation at the site of injury.^[^ ^138^ ^]^ They demonstrated that CX201 was able to significantly reduce ROS of monocytes in an in vitro hemin‐induced injury model. Furthermore, ROS was reduced in microglia, ultimately decreasing the level of recruited macrophages to the site of injury, which is typical in post‐TBI inflammation. Furthermore, the in vivo experiment demonstrated that the treatment led to decreased levels of neutrophil and monocyte recruitment to the injury site, suggesting a diminished inflammatory response compared to the control groups. Finally, CX201 was able to help recover functionality in the brain by enhancing neurogenesis, neurite growth, and synaptic plasticity via the activation of the AMPK‐PKC‐CBP pathway and increased expression of growth‐associated protein‐32.

### 3.5 Carbon Nanomaterials

Carbon nanomaterials (CNMs) are emerging nanomaterials that offer a promising approach to drug delivery, tissue regeneration, and biological sensing.^[^ ^139^ ^]^ Specifically, CNMs such as carbon dots (CDs) are one of the most promising candidates for CNS application due to their ability to penetrate the BBB.^[^ ^140^ ^]^ The characteristics of CDs, tuned within their design, offer high biocompatibility, nontoxicity, excellent photoluminescence, and nanoscale size.^[^ ^141^ ^]^ The components, materials, and methods utilized to synthesize CDs and other CNMs are widely versatile and often depend on the application.

#### 3.5.1 Glioblastoma

Since their discovery in 2004, CDs have been of great interest to scientists for applications in biosensing, bioimaging, and drug delivery. Although most reported biological applications of CDs are in cell imaging and sensing, only a few studies have demonstrated the therapeutic use of CDs.^[^ ^142^ ^]^ In the context of glioblastoma, curcumin, a polyphenolic phytochemical derived from turmeric, has been emphasized as a therapeutic due to intrinsic antioxidant and anti‐inflammatory properties.^[^ ^143^ ^]^ Despite the confirmed anticancer activity of curcumin, its poor solubility, low absorption, rapid metabolism, and clearance present hurdles in its potential use as a therapeutic. In 2021, Sharma et al. prepared curcumin CDs (CurCDs) from curcumin and ethylenediamine by hydrothermal synthesis.^[^ ^144^ ^]^ Synthesized CurCDs were around 4 nm in diameter and possessed superior water solubility and photoluminescence compared to free curcumin. CurCDs had an intrinsic bio‐safe and cancer‐selective property when administered to glioblastoma cells (C6s) and human dermal fibroblasts (HDFs). Treatment of free curcumin showed anticancer properties in the viability of C6 cells and became cytotoxic in HDFs at higher concentrations (250 μg mL^−1^). On the other hand, CurCDs displayed superior anticancer behavior in C6 cells and were noncytotoxic to HDFs up at concentrations as high as 500 μg mL^−1^. Glioma cells have an ability to migrate and invade healthy tissue, resulting in the spread of the cancer. Curcumin has been reported to inhibit glioblastoma cell migration, and the ability of CurCDs to illicit this property was probed with a scratch assay.^[^ ^145^ ^]^ The ability of CurCDs to inhibit glioma C6 cell migration was similar to that of curcumin, with both treatments preventing migration in comparison to untreated conditions. Several more in vitro assays were conducted to probe the apoptotic effect, ROS‐inducing properties, and the effect on cell cytoskeleton of CurCDs in C6 cells in comparison to curcumin. Finally, the biocompatibility of CurCDs was investigated using Zebrafish embryos as a bridge between in vitro and in vivo studies. After 24 h postadministration, embryos treated with CurCDs appeared to have development similar to the untreated control, whereas curcumin treatments cause delayed development and death at higher concentrations. Furthermore, these results confirmed the biocompatibility of CurCD, as well as the improved water solubility, stability, and anticancer properties compared to curcumin.

#### 3.5.2 Neurodegenerative Disease

Recently, graphene CDs (GCDs) were reported to inhibit the fibrillization of PD‐associated amyloid α‐synuclein.^[^ ^146^ ^]^ The synthesized GCDs were able to interact directly with mature fibrils and trigger their disaggregation. Since then, researchers in the field have been probing GCD and CD compositions to aid in neurodegenerative fibrillation inhibition. In 2021, Guerrero et al. synthesized CDs from Na‐citrate as a carbon source through hydrothermal methods to illicit antiamyloidogenic effects in the context of neurodegenerative diseases.^[^ ^147^ ^]^ In this study, investigators used hen‐egg white lysozyme (HEWL) as a model amyloidogenic protein. Fibrils formed were stained by thioflavin T (ThT) staining, resulting in HEWL serving as a standard for studying the amyloidogenesis of other proteins such as Aβ, tau, α‐synuclein, and mutant HTT. Synthesized Na‐citrate CDs were about 2.7 nm in diameter, with high water solubility and stability, supporting their ability to diffuse through the BBB. A pulse‐chase amyloid fibril‐forming assay was conducted to probe the ability of Na‐citrate CDs to inhibit amyloidogenic trajectories. In this assay, CDs were mixed with HEWL in a solution at different timepoints, probing the ability to halt fibrillation at the respective stage. ThT was used to detect large fibrils, in which it specifically stains. The synthesized Na‐citrate CDs effectively inhibited amyloidogenic mechanisms from the point of injection into the HEWL solution. When incorporated from the start, no ThT fluorescence was observed, suggesting maximum inhibition of fibril formation. Finally, the Na‐citrate CDs were treated to SH‐SY5Y neuroblastoma cells to investigate cytotoxic effects. No significant loss in cell viability was observed at concentrations as high as 5 mg mL^−1^.

**Figure 10:**
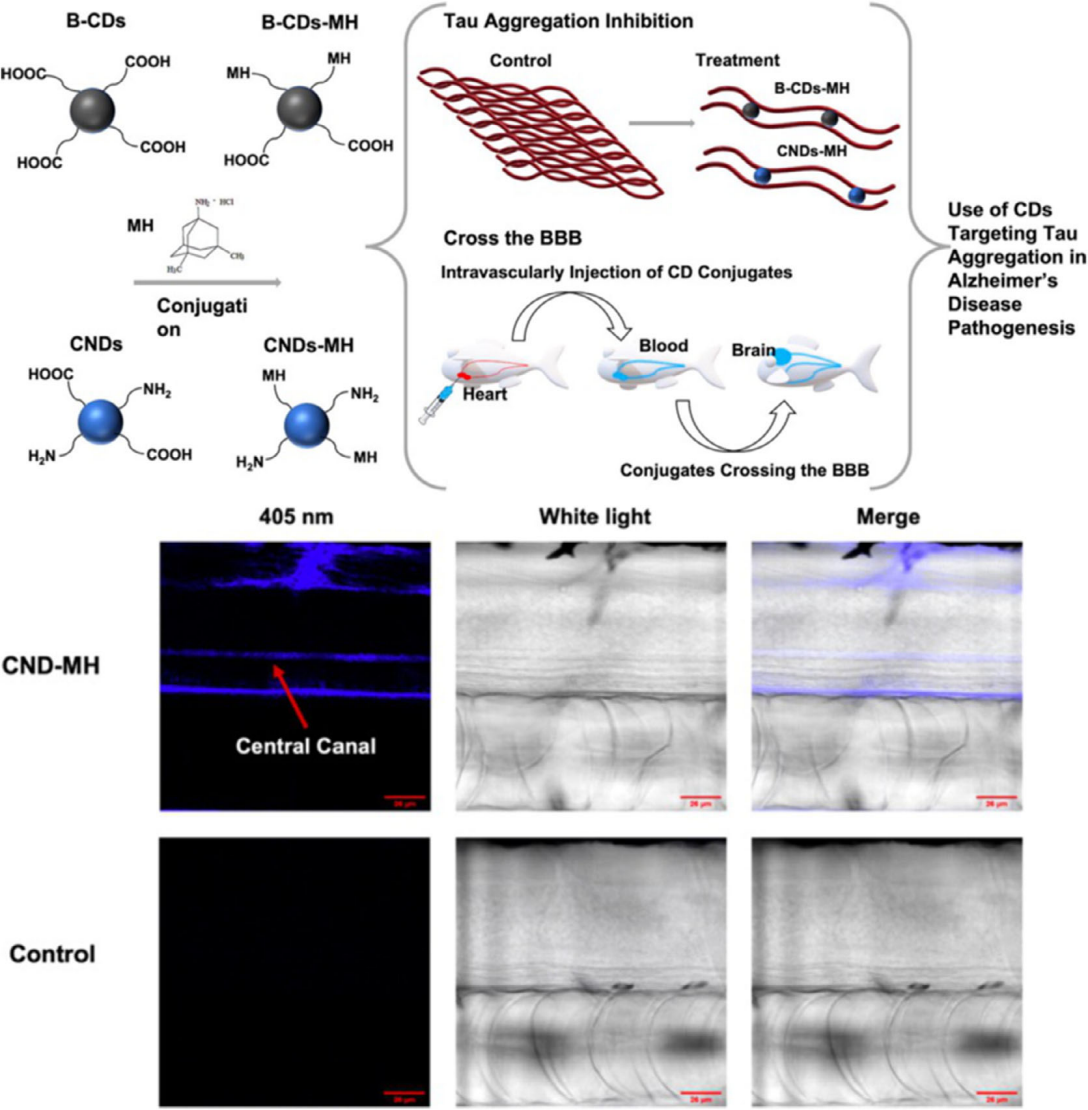
Schematic for the conjugation of memantine onto CNDs, with the proposed mechanism of action (top). The CNDs were imaged in vivo using a zebrafish model, showing the localization and transcytosis of the therapeutic through the BBB. Reproduced and adapted under the Creative Commons Attribution (CC BY) license.^[^ ^148^ ^]^

In a related study in 2022, Zhang et al. synthesized carbon nitride dots (CNDs) conjugated with MH to disaggregate tau, the pathological hallmark of AD (**Figure** **10**).^[^ ^148^ ^]^ Mainly used for the disaggregation of tau, MH is the first US FDA‐approved drug for the treatment of AD. It was found to improve patients’ cognition and abilities to perform daily activities.^[^ ^149^ ^]^ Utilizing CDs as a drug NC, Zhang hypothesized that the efficacy of MH may be enhanced by increasing the delivery efficiency to the brain. CNDs were synthesized by hydrothermal means and MH was conjugated to the surface using EDC/NHS CD (MH‐CD). With an average diameter of 2.2 nm, the synthesized CDs were an ideal size for BBB passive transport.^[^ ^150^ ^]^ Initial tau‐aggregation inhibition assays were performed to investigate the efficiency of the therapeutic method of disaggregating tau. Solutions of tau protein were made with DTT and heparin (to induce aggregation) before incubating with various concentrations of MH‐CDs. Compared to free MH, MH‐CDs possessed far superior antiaggregation properties, as indicated by relative ThT fluorescence. The MH‐CDs were calculated to have IC_50_ values of 1.6 ± 1.5 μg mL^−1^, the typical range of small‐molecule drug candidates. Finally, the ability of MH‐CDs to cross the BBB was investigated in wild‐type zebrafish larvae. Under corresponding excitation wavelength, the blue photoluminescence in the central canal of the spinal cord was indicative of the presence of MH‐CDs, but also passage through the BBB. CDs and other carbon‐based nanomaterials remain a promising and dynamic therapeutic for neurodegenerative diseases, as reported by the findings discussed above.

#### 3.5.3 Ischemic Stroke

CDs have been used more recently in bioapplications of CNS‐related diseases due to their nanosize, high stability, tunability, and ability to diffuse passively through the BBB. Ischemic stroke, often characterized by the lack of blood flow to a region of the brain due to a clot, poses a serious threat to the deoxygenated tissue, causing severe damage. However, while ischemia causes major tissue damage, an influx of free radicals with restored blood promotes extensive inflammatory responses.^[^ ^151^ ^]^ In 2021, Zhang et al. designed a new species of CDs from *Crinis Carbonisatus* for the neuroprotective effect and treatment of ischemic stroke.^[^ ^152^ ^]^ *Crinis Carbonisatus* (CrCi) is a traditional Chinese medicinal material produced from carbonized hair of a healthy human after washing, drying, and calcining. Documented from classic Chinese medical books more than 2000 years ago, CrCi could be used to treat various diseases such as hemorrhage, epilepsy, and stroke. The CrCi‐CDs were synthesized by carbonization in a muffle furnace, before purification in deionized water, yielding 3.2–8.8 nm NPs. To investigate the neuroprotective effects of the CrCi‐CDs, the CDs were treated to MCAO rats, and brain infarction volumes were compared between conditions. Compared with the vehicle group, the CrCi‐CDs had a dose‐dependent inverse correlation with brain infarction volume, significantly decreasing at high concentrations. Additionally, the levels of Evans blue in the ischemic hemispheres, the concentration of inflammatory cytokines, and the neurotransmitter levels in the brain were all measured. Overall, CrCi‐CDs significantly reduced the volume of ischemic lesions and BBB permeability, improved neurological deficits, and decreased the relative levels of TNF‐α and IL‐6 in MCAO rats.

#### 3.5.4 Traumatic Brain Injury

**Figure 11:**
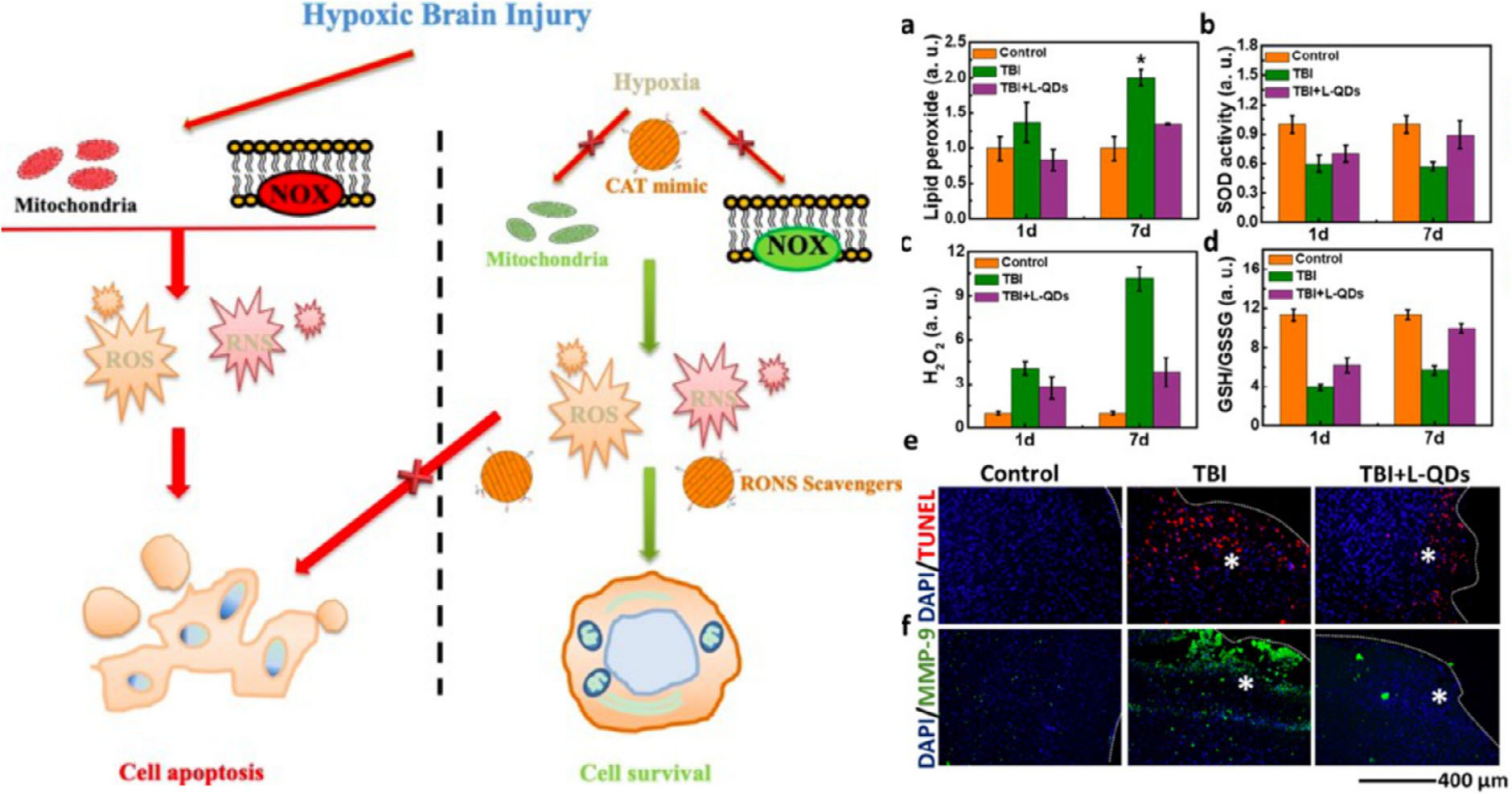
Catalase‐like CDs polymerized by L‐lysine for the decomposition of H_2_O_2_ and liberation of O_2_ gas in the treatment of TBI. A schematic showing the induced cell death from ROS production after TBI is compared to that in the presence of the L‐CDs (left). For a–d (top), H_2_O_2_, lipid peroxidase, SOD, and GSH/GSSG ratios were quantified after 1 and 7 days in the brain of TBI mice. e,f) The presence of proinflammatory, apoptotic, and neuronal biomarkers related to TBI was also imaged via immunostaining. Reproduced and adapted under the Creative Commons Attribution (CC BY) license.^[^ ^153^ ^]^

In TBI, ROS and reactive nitrogen species (RNS) play a crucial role in propagating free radical‐induced oxidative stress. The radicals produced subsequently react with proteins, lipids, and DNA, leading to dysfunction, misfolded proteins, lipid peroxidation, and even cellular apoptosis. To ameliorate this issue, Li et al. designed catalase‐like CDs polymerized by L‐lysine (L‐CDs) for the decomposition of hydrogen peroxide and subsequent liberation of oxygen gas in a TBI in vivo model (**Figure** **11**).^[^ ^153^ ^]^ L‐CDs synthesized by Li and company were prepared by microwave irradiation, with a size of roughly 2.5 nm. Both the catalase activity and RNS scavenging activity were probed for L‐CDs. Catalase activity was probed by UV–vis analysis overtime, in the presence of hydrogen peroxide, displaying proficient ability for peroxide conversion into oxygen gas. For RNS, electron spin resonance (ESR) was utilized to probe the scavenging ability of L‐CDs. ESR analysis showed that after 90 min of the reaction, the removal efficiency reached 84.4%, showing the excellent ability of L‐CDs to remove RNS. Finally, in vivo effects of L‐CDs were tested in TBI mouse models. In many cases of TBI, SOD levels are often reduced due to oxidative stress and activation of immune cells. Upon treatment with L‐CDs via intravenous injection, the activity of SOD in TBI + L‐CD groups was significantly increased. Additionally, L‐CD conditions increased the ratio of GSH/oxidized‐GSH (GSSG) from abnormally reduced levels, indicative that L‐CDs improved the activity of endogenous enzymes to promote the return of GSH and GSSG to normal levels. In all, the oxidative stress indicators and inflammatory responses induced by hypoxic brain injury show an improvement under L‐CD intravenous injection. Additionally, motor function and spatial memory of TBI mice were promoted to an appreciable extent by L‐CD treatment, providing a new insight into the therapy of hypoxic brain injury.

### 3.6 Biomimetic Nanomaterials

Biomimetic nanomaterials include a variety of nanomaterials, such as extracellular vesicles (EVs), cell membrane‐based particles, protein and peptide‐based NPs, as well as RNA‐based NPs.^[^ ^154^ ^]^ EVs have emerged as a promising class of biomimetic NPs for enhancing regeneration following CNS injuries. They comprise a lipid bilayer containing proteins, nucleic acids, and metabolites, which have therapeutic efficacy in resolving inflammation and encouraging neuronal proliferation and differentiation.^[^ ^155^ ^]^

#### 3.6.1 Glioblastoma

**Figure 12:**
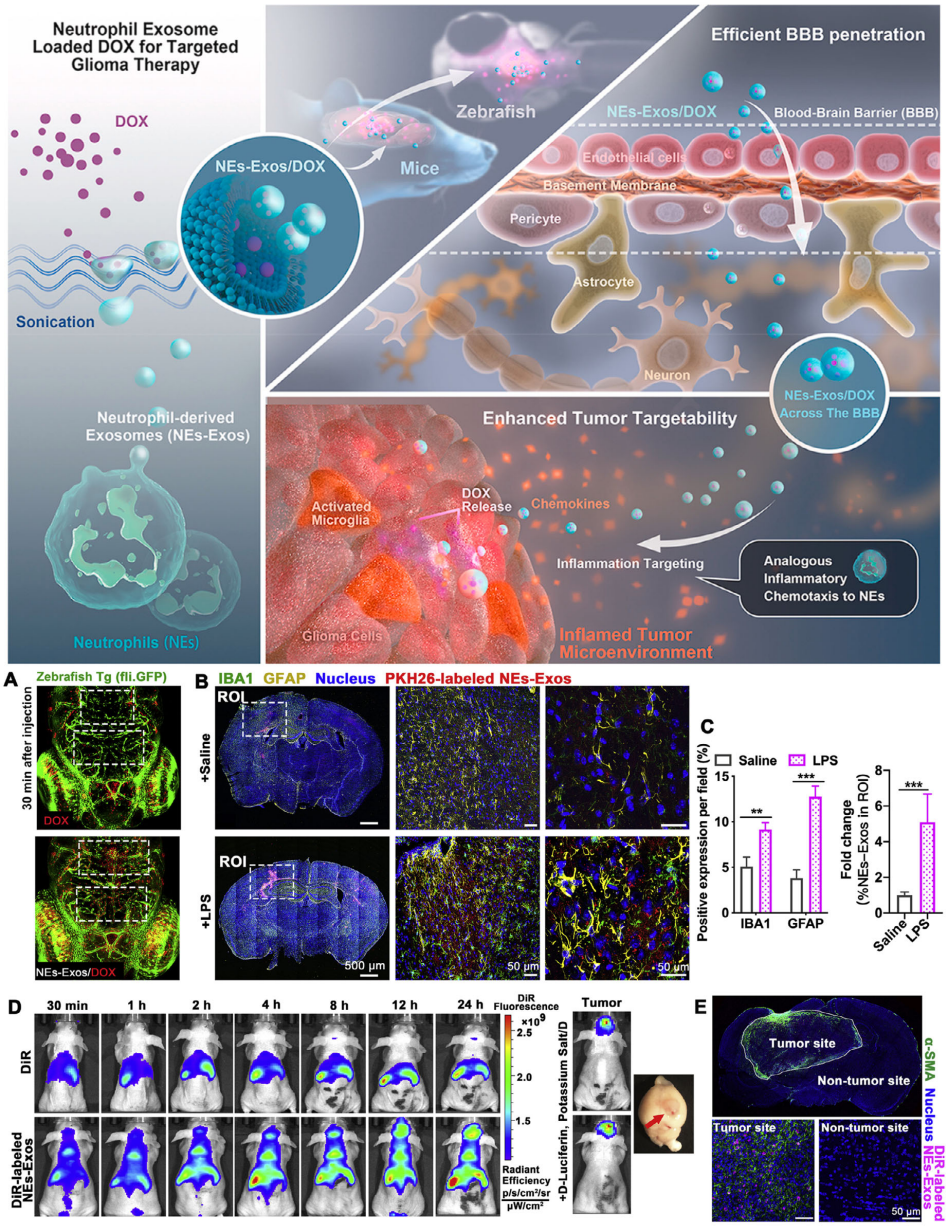
Inflammatory tumor microenvironment‐responsive neutrophil exosomes‐based drug delivery system for targeted glioma therapy. A schematic illustration for loading DOX and improving antiglioma therapeutic outcome with a BBBBB crossing and inflammatory stimuli‐responsive NEs‐Exos drug delivery system (top). A) Confocal images of DOX and NEs‐Exos/DOX crossing the zebrafish BBB at 30 min. B) In vivo distribution of PKH26‐labeled NEs‐Exos (red) in brain with LPS‐induced inflammation. C) Quantification of the fluorescence of IBA1, GFAP, and PKH26‐labeled NEs‐Exos. D) Real‐time fluorescence tracking of DiR and DiR‐labeled NEs‐Exos in C6‐Luc glioma‐bearing mice. E) Representative fluorescence images of DiR‐labeled NEs‐Exo (purple) and α‐SMA (green) in brain tumor at 24 h after administration. Reproduced and adapted under the Creative Commons Attribution (CC BY) license.^[^ ^156^ ^]^

In general, the tumor microenvironment has been characterized by persistent inflammation and is often perceived as a “wound that does not heal.” Because of this, more complex and specific targeting of the local site of inflammation has been investigated through biomimetic NPs, including modified exosomes and cell membrane‐coated NPs. Neutrophils can be effectively recruited to sites of inflammation and also possess an intrinsic ability to traffic the CNS, successfully crossing an intact BBB. Furthermore, exosomes derived from neutrophils are often hypothesized to reflect the properties of their parent cell. In light of this, Wang et al. developed neutrophil‐derived exosomes loaded with DOX (NEs‐Exos/DOX) for delivery across the BBB^[^ ^156^ ^]^ (**Figure** **12**). Mainly focusing on the ability to penetrate the BBB and tumor targetability, several studies were employed including in vitro BBB transwell assays, in vivo zebrafish and mouse brain inflammatory examinations, and in situ antiglioma studies. For preparation, NEs‐Exos were isolated from neutrophils following sequential ultracentrifugation methodology and were loaded with DOX via sonication. Drug loading efficiency and encapsulation efficiency were calculated to be 6.51 ± 0.3% and 13.71 ± 0.7%, respectively. Endocytosis of NEs‐Exos/DOX was investigated to determine the mechanism of internalization, which was experimentally shown to be clathrin dependent. Furthermore, NEs‐Exos/DOX were able to successfully cross the BBB in both in vitro transwell models and in vivo, utilizing RMT as determined by its clathrin‐endocytic dependency. In each in vivo model utilized, fluorescently labeled NEs‐Exos/DOX was able to effectively localize at the tumor site as monitored by real‐time fluorescent tracking, reflecting the inherent neutrophil‐like targeting ability. Finally, the therapeutic value of the treatment was observed using glioma bioluminescence in C6‐Luc glioma‐bearing mice after NEs‐Exos/DOX, DOX, and saline treatment. After treatment, fluorescence quantification displayed 3.52‐ and 2.74‐fold lower intensity in NEs‐Exos/DOX than in saline and DOX groups, respectively. Ultimately, NEs‐Exos/DOX‐treated mice observed a 50% survival rate increase to 27 days, compared with 19 days for saline and 22 days for free DOX.

Cell membrane‐based NPs are a recent development that are feasible to use for therapeutic delivery and disease detection.^[^ ^157^ ^]^ In the context of anticancer treatment, various surface groups of the membranes facilitate prolonged, systematic retention time and reduced immunorecognition, subsequently improving drug accumulation in tumor sites due to EPR effect. Chai et al. designed red blood cell (RBC) membrane‐coated DTX NCs functionalized with c(RGDyK), a tumor‐targeting ligand, for anticancer drug delivery to the brain.^[^ ^158^ ^]^ To prepare RBC‐NC(DTX), RBC membranes were isolated from RBCs and DTX NCs were synthesized via filming‐rehydration methodology. For tumor targeting, c(RGDyK) peptide was incorporated onto the RBC membrane through streptavidin–biotin conjugation chemistry. To assemble the combined drug delivery system, DTX NCs and modified RBC membranes were mixed and sonicated to induce fusion and overall assembly of RBC‐NC(DTX). Initially, in vivo toxicity tests were performed to ensure the targetability and safety of the system regarding off‐target effects. Both RBC‐NC(DTX and RGD‐RBC‐NC(DTX) treatments showed negligible organ damage in vivo, similar to that of control conditions. As hypothesized by the investigators, the in vivo biodistribution and overall antitumor efficacy were superior for RGD‐RBC‐NC(DTX) in relation to free‐drug delivery. Both RGD‐RBC‐NC(DTX) and RBC‐NC(DTX) showed significantly higher tumor accumulation than free‐drug conditions after 2 and 24 h. Furthermore, antitumor studies showed excellent tumor growth inhibition for both RGD‐RBC‐NC(DTX) and RBC‐NC(DTX) conditions, measured every 2 days for 44 days post administration.

Similar to that of cell membrane‐coated NPs, other biomacromolecules have been used to coat NP delivery systems for biomimetic properties. Specifically, many investigators have used lipoprotein‐modified NPs for this purpose by mimicking the endogenous shape and structure of individual lipoproteins, ultimately evading immune cell phagocytosis. Huang et al. encapsulated siRNA‐loaded calcium phosphate (CaP) in high‐density lipoprotein (HDL) for targeted delivery of activating transcription factor‐5 (ATF5) siRNA past the BBB for glioblastoma treatment.^[^ ^159^ ^]^ Taking advantage of Ras activation in cancer cells for enhanced micropinocytosis, ApoE‐rHDL was used as the drug delivery NC containing a CaP core, loaded with ATF5 siRNA for the silencing of the antiapoptotic transcription factor in glioblastoma. In general, ATF5‐CaPs were prepared via water‐in‐oil microemulsion methodology with the inclusion of select lipids to encourage subsequent lipoprotein interactions. Furthermore, the ATF5‐CaP‐LNCs were incubated with ApoE3 to afford ATF5‐CaP‐rHDL. Initially, in vitro studies were performed to investigate and confirm micropinocytosis‐mediated cellular uptake, facilitated by surface‐bound ApoE. Additionally, this endocytosis was based primarily on Ras activation, which was also investigated. Several Ras‐activated and Ras‐inactivated cell lines were treated with ATF5‐CaP‐rHDL, which displayed stark differences in the cellular internalization of the NPs. In general, Ras‐activated cell lines showed significantly more NP uptake in relation to Ras‐inactivated cell lines, strongly suggesting Ras activation‐dependent micropinocytosis as the mechanism for targeted delivery of ATF5‐CaP‐rHDL. To investigate the tumor‐targeting ability of ATF5‐CaP‐rHDL in vivo, real‐time near‐infrared fluorescent tracking was performed by DiR‐labeling. Overall, ATF5‐CaP‐rHDL showed significantly higher BBB transcytosis and tumor accumulation in relation to ATF5‐CaP‐LNC, supporting Ras‐mediated micropinocytosis. Finally, antiglioblastoma activity was studied in vivo using C6 glioblastoma mice, displaying significant differences in survival rates between treatment groups. Over 26 days, the survival rate of the saline, NC‐CaP‐rHDL, ATF5‐CaP‐LNC, and ATF5‐CaP‐rHDL‐treated animals was 0, 11, 48, and 100%, respectively. Likewise, the mean survival of those mice administered with the aforementioned treatments was 18, 21, 26, and 37 days, respectively.

#### 3.6.2 Neurodegenerative Disease

Qi et al. developed quercetin‐loaded exosomes in order to improve the efficiency of brain delivery of quercetin which has been shown to regulate tau phosphorylation and is being studied to treat AD.^[^ ^160^ ^]^ The exosomes led to functional improvements in an okadaic acid‐induced AD mouse model such as improved memory and spatial learning ability as well as showed improved effects over therapeutic delivery without encapsulation in exosomes. The exosomes loaded with quercetin also led to an increase in neuroprotection partially through inhibition of neurofibrillary tangle formation. The exosomes also increased the uptake of quercetin into the cerebrum by 2.5‐fold and the uptake into the cerebellum 1.5‐fold. The work by Qi et al. showed the potential of exosomes for enhancing therapeutic uptake to the brain for effects on AD. Chen et al. isolated and characterized human umbilical cord mesenchymal (hucMSC)‐derived exosomes for treatment using a PD rat model.^[^ ^161^ ^]^ During in vivo testing, neurons from the control disease model were distorted and shrunken, while neurons from the exosome‐treated rat had significantly improved, and imaging showed multipolar neurons. This is due to the growth factors, cytokines, and chemokines released from hucMSCs, which have the potential for neuroprotection, neurodifferentiation, and reduction of apoptosis. The therapeutic target was the substantia nigra past the BBB, so Chen et al. tested dye‐labeled exosome uptake to the brain by injecting them with a chemical to induce α‐synuclein aggregation. The control was saline injected with the same chemical, and confocal microscopy showed higher uptake into the brain of the fluorescence with the exosomes over the control. Therefore, Chen et al. determined that exosomes were able to cross the BBB. They also saw that exosome treatment led to a decrease in dopaminergic neuronal apoptosis and an increase in dopamine levels as well as related metabolites. Outside of exosomes, lipoproteins have also shown promise for the treatment of neurodegenerative diseases. Song et al. combined lipid‐free ApoE3 and phospholipid carriers to create ApoE3‐rHDL, which has the potential for binding to AB and facilitating its degradation.^[^ ^162^ ^]^ ApoE expression in the BBB allows for entry to the brain through RMT as well. One hour after injection, 80% of the Apoe3‐HDL accumulated in the brain parenchyma, and it increased to 94.6% +/− 1.15 after 24 h. Furthermore, in terms of therapeutic effects, ApoE3‐rHDL significantly increased liposomal colocalization of Aβ in astrocytes and microglia. However, the Aβ levels intracellularly decreased drastically, determined by detection through an enzyme‐linked immunosorbent assay (ELISA) assay. Therefore, the lipoprotein was able to cross the BBB and has the potential for the treatment of AD.

#### 3.6.3 Ischemic Stroke

In regard to the treatment of ischemic stroke, it is well understood that the window for treatment is very narrow (<4.5 h). Because of this, NP design strategies for evading the immune system are of the upmost importance when considering therapeutic bioavailability and overall efficacy. Furthermore, targetability also plays a crucial role in therapeutic efficacy and can significantly influence overall bioavailability and retention at the site of inflammation. Tian et al. achieved targeted delivery of c(RGDyK)‐modified exosomes loaded with curcumin to the brain for ischemic stroke.^[^ ^163^ ^]^ In general, c(RGDyK) has been indicated to be a targeting ligand for the ischemic brain, and curcumin, a natural polyphenol, has been highlighted for its anti‐inflammatory and ROS‐scavenging properties. For preparation, exosomes were isolated from bone marrow‐derived MSCs via ultracentrifugation methodology. Conjugation techniques utilizing DBCO‐NHS chemistry were used to conjugate c(RGDyK) to the surface of the exosomes to afford cRGD‐Exo. Additionally, curcumin loading was facilitated by mixing cRGD‐Exo and curcumin at a 6:1 ratio followed by successive centrifugation techniques to remove nonloaded free curcumin, affording cRGD‐Exo‐cur. In vivo, the inhibition of postischemia proinflammatory cytokine signaling by cRGD‐Exo‐cur was investigated. Mice were injected with each treatment condition via the tail vein 12 h after MCAO/R. They were then evaluated at 24 and 36 h postreperfusion. Microglia were isolated from the peri‐infarct area and analyzed by qPCR, Western blot, and fluorescent immunostaining. Ultimately, mRNA levels for TNF‐a, IL‐1B, and IL‐6 were significantly downregulated in cRGD‐Exo‐cur conditions in contrast to PBS, Exosomes, cRGD‐Exo, and free curcumin conditions. Likewise, Western blot analysis reflected the qPCR results, showing relative significance between groups regarding protein expression levels of the same cytokines. Finally, investigators confirmed BBB transcytosis and microglial uptake through brain‐slice image analysis, showing exosomes trapped in brain cells upon intravenous administration 12 h after reperfusion.

Due to the proinflammatory environment of an ischemic lesion, usually governed and exacerbated by high levels of ROS, investigators have utilized nanozymes to reduce the oxidative stress propagated to neighboring cells over time. Additionally, combinatorial approaches using nanozymes with cell membrane‐coated NPs have been of great interest recently. In 2023, Dong et al. designed a homing peptide‐modified neutrophil membrane biomimetic NP in response to ROS and inflammatory microenvironment for precise targeted treatment of ischemic stroke.^[^ ^164^ ^]^ The drug delivery system, coined SHp‐NM@Edv/RCD (SNM‐NPs), consisted of a poly‐ß‐cyclodextrin core (RCD) condensed by ROS‐sensitive phenylboronic acid pinacol esters. Separately, SHp, a peptide able to specifically target the apoptotic nerve cells at the site of stroke, was conjugated to a DSPE‐PEG2000 chain to afford SHp‐PEG‐DSPE for integration into the system. Additionally, neutrophils membranes (NM) were isolated from bone marrow‐derived neutrophils. Finally, the RCDs, SHp‐PEG‐DSPE, and neutrophil membranes were mixed at a specific ratio to afford SNM‐NPs. Investigators utilized C57BL/6 mice and MCAO/R‐treated rats to probe the ability of SNM‐NPs to cross the BBB and target the site of CIRI. Using DiR‐loaded NPs for in vivo fluorescence imaging, three conditions were tested against a saline control: RCD‐NPs/DiR, NM‐NPs/DiR, and SNM‐NPs/DiR. Following tail vein injection after 24 h, RCD‐NPs showed equivalent fluorescence intensity between the ischemic and normal hemispheres of the brain, whereas the fluorescent signals of NM‐NPs/DiR and SNM‐NPs/DiR mainly aggregated in the ischemic hemisphere, ≈1.47‐fold and 3.68‐fold higher, respectively. Once BBB transcytosis was confirmed, the effective repair of damaged neurons by SNM‐NPs in vivo was explored. Following therapeutic administration into MCAO/R‐treated rats, RCD‐NPs, NM‐NPs, and SNM‐NPs all significantly reduced the infarct volume to 21.58 ± 0.90%, 17.95 ± 0.59%, and 10.57 ± 0.56% of the brains, respectively (control saline condition afforded infarct volume to 38.24 ± 2.31% of the brain).

#### 3.6.4 Traumatic Brain Injury

In a recent study, EVs were isolated from neural stem cells (NSCs) and validated in vivo for their ability to mediate neuroprotection.^[^ ^165^ ^]^ The animals treated with the NSC‐EVs exhibited a noticeable decrease in lesion area. The EVs also modulated the neuroinflammatory microenvironment by reducing GFAP expression, but the exact mechanism of the anti‐inflammatory effect is not well understood. EVs also contain microRNAs, which can enhance neurogenesis and neuronal differentiation, as evidenced by the significantly increased amount of nestin compared to the sham group. These beneficial effects accumulated in improved behavioral recovery following injury. Animals treated with the EVs exhibited fewer left hind limb faults, indicating the animals recovered motor function affected by injury. Overall, NSC‐derived EVs showed significant recovery following TBI. However, the delivery method of choice was intravenous administration, which is potentially hazardous due to the lack of available biodistribution data and potential off‐target effects on other organs.^[^ ^166^ ^]^ Local injection to the injury site is preferred to reduce off‐target toxicity. Hydrogels have also been shown to effectively encapsulate and deliver the EVs to the surrounding neural tissue in a prolonged manner. Li et al recently synthesized an anti‐inflammatory hydrogel doped with EVs from human exfoliated deciduous teeth (SHED) for in vivo reprogramming of microglia following TBI.^[^ ^167^ ^]^ The hydrogel polymer comprises citric acid, 1,8‐octanediol, polyethylene glycol, polyethyleneimine, and gallic acid. The hydrogel can be delivered via injection due to the platform's ability to get at 37°. The hydrogel significantly eliminated free radicals to attenuate LPS‐induced ROS production and downregulated proinflammatory cytokines. The SHED‐derived EVs were found to undergo a gradual release from the hydrogel over 21 days, with higher levels of release observed at lower pH. When tested in vivo, the platform showed a remarkable recovery, as evidenced by improved modified neurologic severity score and BBB scoring. Lower levels of injury site volume were also observed. Another method to limit off‐target effects and maximize therapeutic efficacy includes engineering the EV surface with a neuron‐specific targeting ligand. In a paper by Haroon et al, EVs were engineered with an RVG29 peptide to target neurons via intravenous injection following TBI.^[^ ^168^ ^]^ BV2‐derived EVs were decorated with DBCO to undergo a copper‐free CD reaction with the azido RVG29 peptide. Another peptide, NR2B9c, was then loaded into the EV as a neuroprotective agent. The EVs had little effect on cell viability while drastically reducing ROS. In vivo, biodistribution assays showed a majority of the accumulation in the brain and the liver. The EVs proved incredibly efficacious by significantly reducing lesion volume and modifying neurological severity scores. The study demonstrates the ability to undergo cell‐selective targeting of EVs to maximize therapeutic efficacy while limiting off‐target effects.

### 3.7 Clinical Advancements in Nanomaterials for Neurological Diseases

Although the end goal of nanomaterial‐based treatment is to improve therapeutic efficacy in the clinic, the ability of nanomaterials to overcome the BBB in a clinically relevant setting is critical to their use. Some clinical trials have been performed with nanomaterials as therapeutic treatments for neurological diseases although many were discontinued due to low efficacy and off‐target effects.^[^ ^169^ ^]^ In general, NP design must overcome several challenges for use in clinical trials, such as predicting allergic reactions, controlling sustained drug release, consistent ligand attachment for targeting, and manipulating immune system responses.^[^ ^170^ ^]^ With these considerations, there are a few ongoing clinical trials related to nanomaterial treatments for brain‐related diseases.^[^ ^171^ ^]^ APOLLO: Patisiran ALN‐TR02 is an NP assembly consisting of siRNA‐loaded lipid NPs currently in phase three clinical trials for transthyretin‐mediated amyloidosis, a rare neurodegenerative disease, involving the misfolding of transthyretin.^[^ ^172^ ^]^ Recently, the phase three study concluded and results were released.^[^ ^173^ ^]^ There is an upcoming clinical trial set to start in October 2024 on Australasian NP‐mediated magnetically enhanced diffusion for ischemic stroke (AusNanoMed). An external magnet will pull the IONPs through stagnant blood vessels to restore blood flow to the brain.^[^ ^174^ ^]^ There is also an upcoming clinical trial with another IONP called Pulse NanoMed System.^[^ ^175^ ^]^ It utilizes a similar idea as AusNanoMed utilizing an external magnet for particle directing and is tasked with clot removal following an acute ischemic stroke. In the context of intranasal drug administration in Alzheimer‐type dementia, APH‐1105 NPs are in a phase 2 clinical trial and modulate alpha‐secretase. It is being used to treat AD patients with mild‐to‐moderate cognitive impairment.^[^ ^176^ ^]^ The study concludes in December of this year, and as of 2023 was 1 of 187 clinical studies for AD treatment of any therapeutic type.^[^ ^177^ ^]^ Gadolinium‐based NPs are currently being used in a phase 2 clinical trial for guided brain‐directed stereotactic radiation therapy which is set to be completed in 2026.^[^ ^178^ ^]^ By comparing stereotactic radiation in the presence of the NP to standard stereotactic radiation for brain metastasis and brain tumors, they can determine if the NP enhances the accuracy and effectiveness of radiation therapy. Another phase 2 clinical trial was published in 2023 that had studied gold nanocrystals, CNM‐Au8, for PD treatment.^[^ ^179^ ^]^ The surface of CNM‐Au8 nanocrystals catalyzes the oxidation of NADH to NAD^+^ which led to neuroprotection and remyelination in preclinical testing. Furthermore, the phase 2 testing showed that NAD^+^/NADH levels increased by 10.4% after 12 weeks of treatment with CNM‐Au8.

One paper in 2021 explored improving the BBB delivery of siRNA‐conjugated gold NPs for the downregulation of Bcl2L12. This treatment was used in a phase 1/2a clinical trial (2B3‐101) as a treatment for brain metastasis stemming from breast cancer and other cancer types.^[^ ^180^ ^]^ For future clinical research, the need for more efficient NP delivery across the BBB was recognized. For example, GSH‐targeted liposomes were designed and administered using the postinsertion method, which showed a major improvement in brain delivery over the preinsertion method. In this way, the need for further development of current NPs in testing is a testament to the importance of efficient BBB passage for the treatment of these brain diseases.

Targeted delivery of NPs with the methods described in this review have great potential to increase overall clinical translation. The lack of clinical trials to date and the failure of multiple clinical trials can be attributed to off‐target effects and the low efficiency for BBB delivery.^[^ ^181^ ^]^ Therefore, the focus on targeted delivery past the BBB is critical to the development of nanotherapeutics. There are other challenges, however, that are also critical to therapeutic development for clinical application such as mitigating off‐target effects while enhancing therapeutic efficacy. These challenges can be overcome by targeted delivery across the BBB, but other methods, such as systems that selectively release therapeutics due to biophysical cues, should not be overlooked. Combining the methods discussed here with selective drug release has great potential for increasing the clinical feasibility of many nanomaterials used for the treatment of neurological disorders.

## 4 Conclusion and Future Perspectives

Crossing the BBB is a critical hurdle in the development of effective therapies for any disease that is primarily located within the brain. The intricate architecture and highly selective permeability of the BBB are essential for maintaining the delicate microenvironment of the brain, but also present major obstacles for the delivery of therapeutic agents such as drugs, antibodies, and cells. Furthermore, pathological changes in the BBB associated with various disease states can significantly alter its function and permeability, adding to the complexity of designing effective drug delivery strategies that can overcome this formidable barrier.

Due to their unique properties and versatility, nanomaterials have emerged as promising tools for targeted drug delivery across the BBB. The BBB restricts the entry of many otherwise effective drugs into the brain, limiting their accumulation in diseased areas. By integrating nanomaterials that can actively target the BBB, these drugs and other therapeutics can be delivered to the diseased site more effectively. Additionally, many nanomaterials can be designed and tailored to treat a specific disease by taking advantage of stimuli‐responsive drug release and cell targeting.

Polymer and lipid‐based nanomaterials have demonstrated remarkable potential in treating glioblastomas and neurological disorders, benefiting from their biodegradability, sustained drug release properties, and ability to traverse the BBB when appropriately designed. The diverse range of nanomaterial properties offers numerous possibilities for BBB passage, depending on the specific target of interest. Ultrasmall NPs, such as gold nanoclusters and CDs, are particularly well‐suited for BBB crossing due to their tiny size and ability to adopt the properties of surface ligands. Larger NPs, including polymers and liposomes, are ideal for delivering larger therapeutic cargoes, such as antibodies, RNA, and DNA. MNPs have also garnered significant attention due to their inherent ability to be manipulated exogenously through the use of magnetic fields when delivering therapeutic drugs or biologics such as stem cells. Biomimetic NPs, including EVs, lipoproteins, and cell membrane‐based NPs, also have the potential for crossing the BBB and limit the concern of an immune response due to the biological nature of the NPs.

While substantial progress has been made in understanding the mechanisms by which NPs cross the BBB, many unknowns remain and warrant further investigation. For example, in the context of an NP system that has the potential to pass the BBB by utilizing either AMT or RMT, which mechanism is prioritized? Additionally, are there other mechanisms of NP‐based BBB transcytosis that cannot be categorized into AMT, RMT, CMT, or diffusion? Fully understanding how systems work to pass the BBB, such as rabies virus glycoprotein, can allow for better design of NPs for treating neurological diseases and allow for optimization of BBB passage. The ultimate challenge lies in creating a biocompatible agent with high loading efficiency and sustained drug release properties that can effectively cross the BBB for the treatment of diseases. As research in this field continues to advance, it holds great promise for the development of novel nanomaterial‐based therapies that can overcome the formidable obstacle of the BBB and revolutionize the treatment of neurological disorders.

## Conflict of Interest

The authors declare no conflict of interest.

## Author Contributions

**Callan D. McLoughlin**: Conceptualization (lead); Writing—original draft (equal); Writing—review & editing (equal). **Sarah Nevins**: Conceptualization (lead); Writing—original draft (equal); Writing—review & editing (equal). **Joshua B. Stein**: Visualization (lead). **Mehrdad Khakbiz**: Conceptualization (supporting). **Ki‐Bum Lee**: Funding acquisition (lead); Resources (supporting). **Callan McLoughlin** and **Sarah Nevins** contributed equally to this work.
